# Exploring Diagnostic Reliability of CBCT for Vertical Root Fractures: A Systematic Review and Meta-Analytical Approach

**DOI:** 10.1155/ijod/8824867

**Published:** 2025-07-21

**Authors:** Luiz Carlos de Lima Dias-Junior, Diego Leonardo de Souza, Adriana Pinto Bezerra, Marcio Correa, Cleonice da Silveira Teixeira, Eduardo Antunes Bortoluzzi, Lucas da Fonseca Roberti Garcia

**Affiliations:** ^1^Department of Diagnosis and Oral Health, Division of Endodontics, School of Dentistry, University of Louisville, Louisville, Kentucky, USA; ^2^Department of Dentistry, Division of Endodontics, Federal University of Santa Catarina (UFSC), Florianópolis, Santa Catarina, Brazil

**Keywords:** artifacts, cone-beam computed tomography, diagnosis, meta-analysis, systematic review, tooth fractures

## Abstract

This systematic review investigated the different factors associated with the diagnostic accuracy of vertical root fractures (VRFs) with cone-beam computed tomography (CBCT) scans, assessed by in vitro studies. Studies were screened from PubMed, Embase, Scopus, Web of Science, and Lilacs, up to May 2025. The included studies assessed the diagnostic accuracy of CBCT scans for laboratory-induced VRFs. The quality assessment of the included studies was performed using the QUADAS-2 tool. Meta-analyses were performed using the bivariate model with random effects to produce summary sensitivity (SSe) and specificity (SSp) with a 95% confidence interval (CI). The influence of confounding factors on the accuracy of CBCT images was investigated by meta-regression models. Covariates were added to the bivariate model to assess the impact on sensitivity, specificity, or both. The quality of evidence of each meta-analysis was assessed using the GRADE approach. One hundred studies were included. Twenty-four studies presented a low risk of bias, 22 moderate risk, and 54 high risk. CBCT scans presented a higher sensitivity for the diagnosis of complete VRFs compared to incomplete fractures. The presence of metal posts impaired both sensitivity and specificity. Smaller voxel sizes favored the detection of VRFs in teeth with metal posts. In laboratory settings, the diagnosis of VRFs by CBCT images is mainly affected by the fracture pattern, presence of intracanal materials, and voxel size.

## 1. Introduction

Vertical root fractures (VRFs) extend along the vertical axis of the tooth root toward the apex [1]. The fractures are longitudinally oriented and might be a partial or complete rupture of the tooth root [1]. This type of fracture is more commonly found in endodontically treated teeth, with a reported prevalence ranging from 10.9% to 31.7% in extracted teeth [2, 3]. Early detection and management of VRFs are essential to avoid unnecessary and inappropriate treatment and minimize undesirable consequences, such as perirradicular bone loss [4].

Accurate diagnosis of VRFs is challenging since they may develop slowly [5]. Clinical examination might provide limited information due to the lack of specific signs and symptoms [5], which might include swelling, increased tooth mobility, tenderness to percussion or biting, or evidence of fracture lines [6]. In addition, the examination of periapical radiographs is limited due to their two-dimensional nature and superimposition of images [7].

Usually, VRFs occur in the buccolingual plane and less commonly in the mesiodistal plane [6]. The presence of intracanal materials (i.e., root filling materials and intracanal retainers) makes it difficult to visualize the vertical fracture line, especially the initial or incomplete ones [8]. There is minimal separation between fragments making diagnosis even more challenging [8]. Pathognomonic signs of VRFs, such as deep and narrow periodontal probing depth or J-shaped radiographic lesions, are often observed at advanced stages, when the root fragments are largely separated [4].

Radiographic evaluation of VRFs may be improved with the use of cone-beam computed tomography (CBCT), due to the possibility of the acquisition of three-dimensional images with micrometric resolution and the absence of overlapping [9]. CBCT scans have been highly recommended in endodontics for the diagnosis of contradictory or nonspecific clinical signs, including in the suspicion of VRFs [7]. However, several technical factors might influence the quality and accuracy of CBCT images, such as voxel and field-of-view (FOV) sizes, tube current, and voltage [10].

Patient-related factors, such as the presence of high-density intracanal materials like gutta-percha and metal posts, can generate beam hardening artifacts, which present as streaking and cupping [10] and may obscure adjacent structures, compromising the detection of VRFs [11, 12]. Sharpness filters and metal artifact reduction (MAR) tools have been developed to reduce the impact of these radiodense materials on diagnostic accuracy and improve CBCT image quality. However, the results from studies that evaluated these tools are still controversial [13–22].

Many factors may influence the VRF diagnosis. This systematic review investigated the different factors associated with the diagnostic accuracy of VRFs by CBCT scans. In vivo studies on VRFs diagnostic accuracy are still scarce, and there is limited information available in the literature, with high methodological heterogeneity. Therefore, only in vitro studies were included, and due to the large number of data available, it was possible to evaluate each factor individually and analyze its influence on the diagnostic accuracy of CBCT images for the detection of VRFs.

## 2. Materials and Methods

### 2.1. Protocol and Registration

The protocol for this systematic review and meta-analysis was registered (CRD42020207094) in the PROSPERO (International Prospective Register of Systematic Reviews; https://www.crd.york.ac.uk/prospero/) database. The review was conducted in full accordance with the Cochrane Handbook for Systematic Reviews of Diagnostic Test Accuracy. Reporting for this review follows the guidelines from the Preferred Reporting Items for Systematic Reviews and Meta-Analyses of Diagnostic Test Accuracy (PRISMA-DTA) [23]. Due to the in vitro nature of the included studies, some of the PRISMA-DTA items were adjusted.

### 2.2. Search Strategy

Electronic searches were conducted up to May 12, 2025, in the following databases: Scopus, Embase, Web of Science, Pubmed, and Lilacs. No restrictions to publication date or language were applied. In addition, the electronic databases ProQuest Dissertations and Theses, LIVIVO, and Google Scholar were also inspected to provide gray literature and unpublished studies. Finally, the references of the included studies were examined to identify possible studies, and experts were consulted for potentially eligible articles as part of the search process. The exact search strategy that was used for each electronic database is presented in detail in Supporting Information 1.

### 2.3. Eligibility Criteria

#### 2.3.1. Inclusion Criteria

The inclusion criteria were developed based on the PIRDS strategy [24], as outlined below:

• Participants (P): human extracted teeth;
• Index test (I): CBCT scans;
• Reference test (R): visual inspection of extracted teeth with or without the aid of magnification, transillumination, and/or stains, and micro-computed tomography (micro-CT);
• Diagnostic (D): VRFs;
• Study design (S): in vitro studies assessing the diagnostic accuracy of VRF with CBCT scans.

#### 2.3.2. Exclusion Criteria

The exclusion criteria were applied as follows:

1. Studies with primary human teeth or animal teeth;
2. Studies that included teeth with incomplete root formation;
3. Studies that did not evaluate CBCT as the index test;
4. Studies that did not investigate the diagnostic accuracy of VRFs;
5. Studies with fracture simulation that are not consistent with the real aspect of VRFs;
6. In vivo studies;
7. Reviews, letters, case reports, and case series.

### 2.4. Study Selection

Study selection was conducted in two phases. In the first phase, two trained reviewers (Luiz Carlos de Lima Dias-Junior and Diego Leonardo de Souza) independently screened titles and abstracts. All records were evaluated based on the inclusion and exclusion criteria, with eligible articles selected for full-text review. Any disagreements were resolved through discussion or by consulting a third reviewer (Adriana Pinto Bezerra). The second phase comprised the full-text analysis of each study. According to the eligibility criteria, the studies were included or excluded from this systematic review. Disagreements between reviewers were settled through discussion, and when necessary, a third author was consulted.

### 2.5. Data Extraction

The data extraction from each study was carried out by two independent authors (Luiz Carlos de Lima Dias-Junior and Diego Leonardo de Souza). The following data were extracted from the studies and inserted into a structured form: name of the first author; year of publication; country of the first author; sample size; type of teeth; method of fracture induction; type of fracture (i.e., complete or incomplete); experimental groups and subgroups; CBCT device; acquisition parameters for the CBCT scans; application of image filters or acquisition algorithms; root canal conditions (i.e., no filling, root canal filling, metal post, fiberglass post, or other); simulation of in vivo conditions (i.e., use of human skull or mandible); diagnostic results in terms of sensitivity and specificity values, and true-positive (TP), false-positive (FP), false-negative (FN), and true-negative (TN) rates; and main conclusions. For studies that did not report the TP, FP, TN, and FN rates, they were calculated based on the sensitivity, specificity, and prevalence values, and sample size, using the calculator in Review Manager 5.4 software (Cochrane Collaboration, Oxford, UK). When there was a lack of information or data in any of the included studies, the reviewers contacted the corresponding author by e-mail. Up to five attempts were made to reach the corresponding author, with 7-day intervals. Disagreements regarding data extraction were resolved by discussion with a third author (Adriana Pinto Bezerra). See Supporting Information 2 for the complete extraction form with all the data collected from the included studies summarized in a table.

### 2.6. Quality Assessment of the Included Studies

The quality of the included studies was independently evaluated by two reviewers (Luiz Carlos de Lima Dias-Junior and Diego Leonardo de Souza). For this systematic review, the Quality Assessment of Diagnostic Accuracy Studies 2 (QUADAS-2) tool [25] was adapted, as certain questions (e.g., patient randomization and the index test threshold) were not applicable.

QUADAS-2 assesses the methodological quality of diagnostic accuracy studies based on four major domains regarding the risk of bias: patient selection, index test, reference standard test, and flow and timing; and three domains regarding the applicability concerns: patient selection, index test, and reference standard test. “Review specific” descriptions of how the QUADAS-2 items were contextualized and implemented in our systematic review are detailed in Supporting Information 3.

In each aspect, if the answer to the leading questions were “yes,” then it was given a “low” risk of bias and applicability concern judgment. If any answer was “unclear,” then it was judged to have “some concerns” regarding the risk of bias and applicability concerns. Similarly, if any of the answers were “no,” the domain was judged as “high” for risk of bias and applicability concerns. To reach an overall judgement, it was considered that if the study presented one or more high-risk domains, it was classified as “high-risk of bias.” If the study presented two or more unclear domains, it was classified as “moderate risk of bias.” If all domains presented low risk, or only one domain presented unclear risk, the study had a “low risk of bias” classification. Discrepancies between reviewers were discussed and settled with the assistance of three experts (Cleonice da Silveira Teixeira, Eduardo Antunes Bortoluzzi, and Lucas da Fonseca Roberti Garcia.).

### 2.7. Quality of Evidence

The quality of the evidence found for each factor regarding its influence on the diagnosis of VRF through CBCT scans, was evaluated according to the standards of the GRADE (Grading of Recommendations, Assessment, Development, and Evaluation), using the GRADEpro GDT application (https://www.gradepro.org). Two authors (Luiz Carlos de Lima Dias-Junior and Adriana Pinto Bezerra) rated the quality of the evidence for each factor, according to the answers regarding five domains: risk of bias, inconsistency, indirectness, imprecision, and publication bias. The authors graded each outcome independently, and the experts (Cleonice da Silveira Teixeira, Eduardo Antunes Bortoluzzi, and Lucas da Fonseca Roberti Garcia) were consulted to resolve differences between reviewers. The GRADE system provided support for recommendations of diagnostic tests or strategies for VRF identification. The quality of evidence presented by the GRADE system may vary from high to very low [26].

### 2.8. Statistical Analysis

Initially, comparative analyses included all studies with relevant data, by direct or indirect evidence. Meta-analyses were performed using the bivariate model with random effects to produce summary sensitivity (SSe) and specificity (SSp), and their respective confidence intervals (CIs) [27]. We investigated the factors associated with the accuracy of CBCT scans by meta-regression, adding covariates to the bivariate model to assess the association with sensitivity or specificity, or both. Significant differences in test performance were evaluated by a likelihood ratio test comparing models with and without covariate terms for sensitivity and specificity. The models were fitted using the “glmer” function in the “lme4” package for R version 4.1.3 software (R Foundation for Statistical Computing, Vienna, Austria. URL: https://www.R-project.org/). For each analysis, summary ROC curves were plotted using Review Manager 5.4 software (Cochrane Collaboration, Oxford, UK). We assessed interstudy heterogeneity through the chi-squared-based *Q*-test and inconsistency index (*I*^2^). A significance level of 5% was adopted for all analyses.

## 3. Results

### 3.1. Study Selection

**Figure 1:**
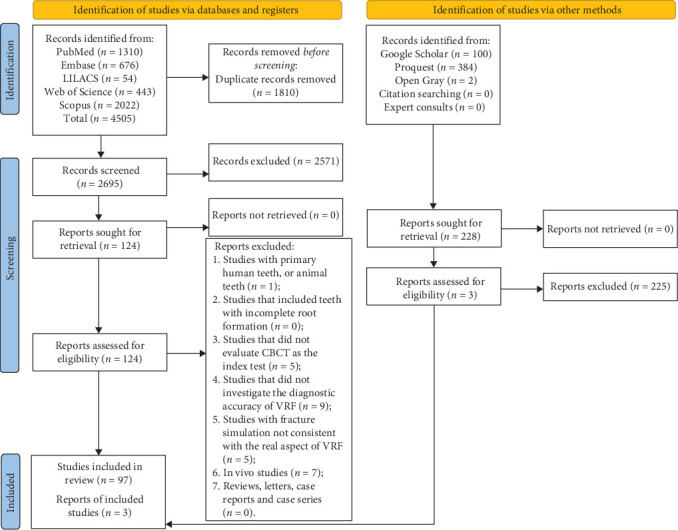
PRISMA 2020 flow diagram.

After electronic searches, we identified 4505 records on the five different databases accessed in this study. Duplicate records were identified, and 1810 studies were removed, resulting in 2695 articles that were screened by a comprehensive evaluation of titles and abstracts (phase 1). For full-text assessment (phase 2), 124 records were retrieved for being considered potentially useful, of which we excluded 27 articles (see the reasons for exclusion in Supporting Information 4), resulting in 97 included studies. An additional search on gray literature, consult of experts, and a reference list of selected studies provided 228 possibly eligible articles, of which three studies met the eligibility criteria after full-text assessment. Thus, 100 articles were selected to answer the questions proposed by this systematic review. See Figure 1 for the detailed process of studies identification, inclusion, and exclusion in the PRISMA 2020 flow diagram [28].

### 3.2. Study Characteristics

The publication years of the included studies ranged from 2009 to 2025. A total of 5808 teeth were analyzed. The methods of VRF included hammer and pin [13, 16, 22, 29–57], hammer and chisel [15, 58–65], bench vise [66], universal testing machine [7, 8, 12, 14, 17–19, 21, 67–110], post or pin turned into the canal [20, 89, 111, 112], and also temperature cycling [113]. Out of the 100 articles, 20 investigated complete fractures [13, 15, 16, 22, 32, 33, 42, 43, 46, 56, 57, 60–64, 70, 73, 81, 93], while 24 studies investigated incomplete fractures [12, 19, 20, 35, 36, 38, 58, 59, 65, 66, 74, 76, 83, 86, 88, 90, 95–97, 102, 106, 110, 111, 113], 21 included both types of fracture [7, 8, 14, 21, 30, 34, 40, 47, 50, 53, 68, 72, 75, 77, 85, 87, 89, 98, 100, 101, 112], and 35 were unclear regarding which type of fracture was produced [17, 29, 31, 37, 39, 41, 44, 45, 48–52, 54, 55, 67, 69–71, 79, 80, 82, 84, 91, 92, 94, 99, 103–105, 107–109, 114, 115]. To simulate in vivo conditions, these teeth were placed in the sockets of dry human mandible or skull [8, 12, 14–19, 21, 29, 35, 37, 38, 41–43, 47, 51–53, 58, 60–63, 68–76, 78, 79, 81–83, 85, 87, 88, 91, 92, 95–100, 102–105, 107–110], ovine mandible [55], gypsum stone blocks [48, 64, 66, 86, 111, 115], bovine rib sockets [13, 20, 22, 31–33, 46, 49, 56], pig mandible [57], acrylic blocks [7, 34, 36, 59, 65, 67, 77, 84, 89, 93, 94, 106, 113], macerated bone artificial sockets [50], and wax models [90].

Investigation groups included teeth with no root canal fillings [8, 12, 14, 16–19, 29, 30, 35, 39, 40, 42–44, 47, 48, 52, 53, 56–65, 69, 73, 74, 78, 79, 82–85, 88, 91, 95, 96, 99, 100, 106–108, 110, 113, 115], gutta-percha with or without root canal sealer [12–16, 20, 22, 29, 30, 32, 33, 35, 36, 38, 39, 41–49, 51, 53, 56, 57, 59–61, 64, 65, 67, 75, 77–81, 83, 85, 87, 88, 92, 96, 97, 100, 101, 104, 106–108, 111, 112], metal posts [7, 12, 14–17, 20, 29, 31, 34–36, 42–45, 47, 50, 53–55, 57, 59, 65, 68, 72, 78, 80, 81, 83–86, 89, 90, 92–94, 96, 99, 100, 102, 103, 105, 108, 109, 111, 114], fiber posts [16, 47, 50, 53, 54, 57, 70, 71, 78, 85, 99, 100, 108], bioceramic root canal filling material [67, 110], and zirconium based root canal filling material [75].

Image acquisition was performed with a variety of CBCT devices set with kilovoltage ranging from 120 to 60 kVp, voxel size from 0.075 to 0.4 mm, and FOV size from 23 × 23 to 4 × 4 cm. In 21 of the included studies, a variety of MAR algorithms were applied during image acquisition [13, 16, 18, 20–22, 49, 56, 68, 74, 76, 86, 93, 102–104, 106, 107, 109, 111, 114]. Image filters were also evaluated. Four studies applied artifact reduction filters to the acquired images [14, 49, 65, 100], and five studies used sharpness filters in the images [15, 17, 19, 46, 109]. The characteristic of each included study is presented in Supporting Information 2.

### 3.3. Quality Assessment

**Figure 2:**
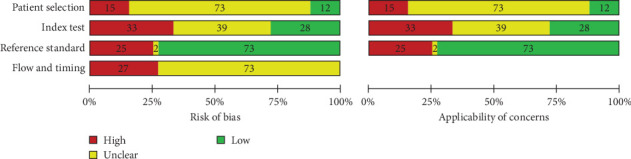
Summarized results of the quality assessment for the included studies.

**Figure 3:**
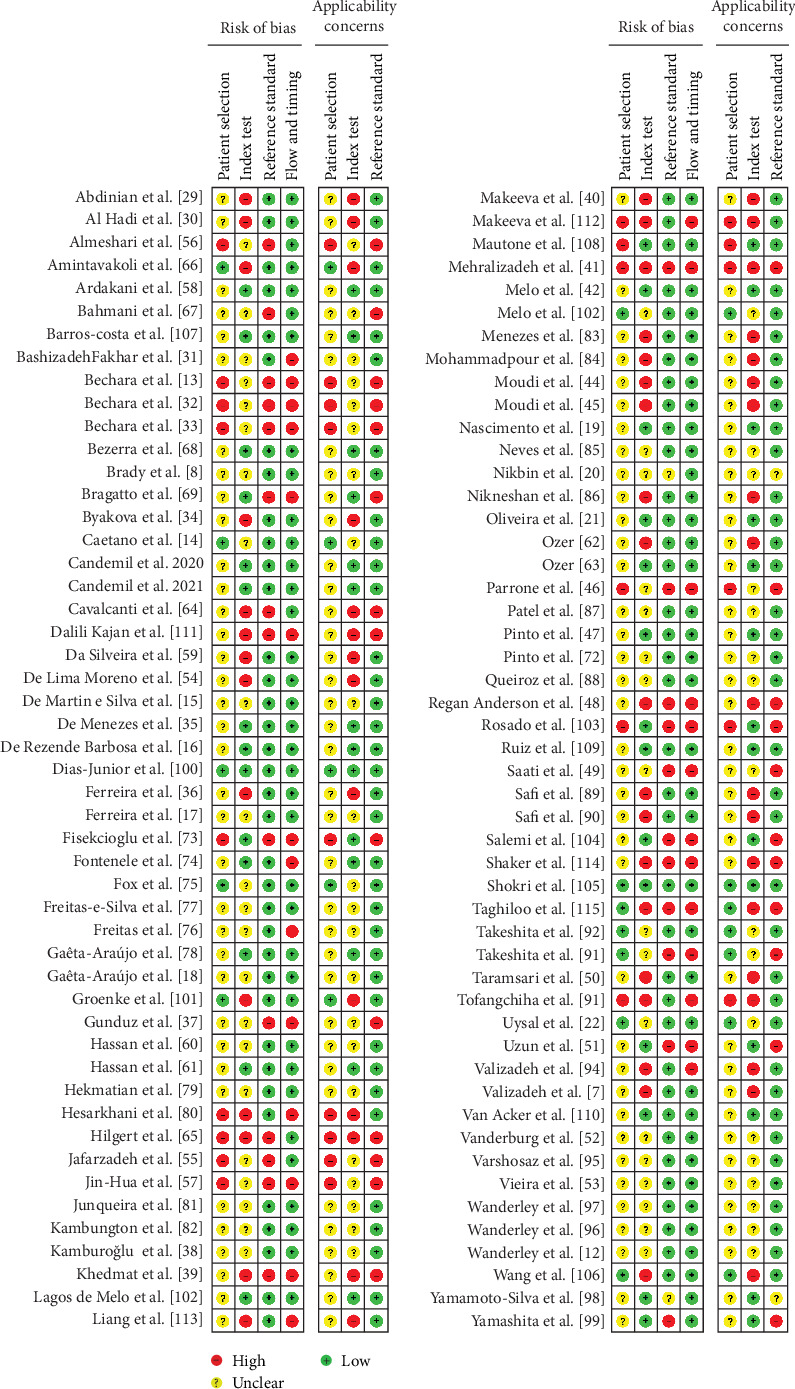
Detailed results of the quality assessment according to the QUADAS-2 appraisal tool for diagnostic accuracy studies.

The summarized results for risk of bias and applicability concerns assessment of the 100 included studies are presented in Figure 2. Overall, only 24 of the included studies presented a low risk of bias and applicability concerns [14, 16, 19, 21, 22, 35, 42, 43, 47, 58, 61, 63, 68, 70, 71, 75, 78, 92, 100, 102, 105, 107, 109, 110]. Twenty-two studies presented a moderate risk of bias due to unclear or no description of sample size calculation and the use of bovine bone and acrylic or gypsum blocks for simulation of in vivo conditions [8, 12, 15, 17, 18, 20, 38, 52, 53, 60, 72, 77, 79, 81, 82, 85, 87, 88, 95–98]. Additionally, the 54 remaining studies presented a high risk of bias, especially due to the lack of baseline evaluation of the teeth [13, 32, 33, 41, 46, 49, 56, 57, 65, 67, 73, 74, 76, 93, 103, 108, 113, 114], issues with index test methodology, such as the absence of in vivo conditions simulation (isolated tooth) and lack of examiner blinding [7, 29–31, 34, 36, 39–41, 44, 45, 48, 50, 51, 59, 62, 65, 66, 80, 83, 84, 86, 89, 90, 93, 94, 101, 106, 108, 111–115]. High risk was also considered when there was no description of the reference test [13, 32, 33, 37, 39, 41, 46, 48, 49, 51, 56, 65, 67, 69, 73, 91, 99, 104, 111, 114, 115], or the reference standard test was performed only on part of the sample (fractured or nonfractured teeth) [13, 24, 31–33, 37, 39, 41, 46, 48, 49, 51, 57, 69, 73, 74, 76, 80, 91, 93, 94, 103, 111–115]. The detailed quality assessment of the included studies is shown in Figure 3.

### 3.4. Synthesis of the Results

The possible factors associated with the diagnostic accuracy of CBCT scans for VRFs identified from the primary studies were fracture pattern (i.e., complete or incomplete); acquisition parameters (FOV and voxel sizes, tube current, tube voltage); intracanal materials; image enhancement filters; MAR algorithms; CBCT device; tooth position within FOV; and the presence of adjacent dental implants. The meta-analyses were performed using only studies that presented low or moderate risk of bias and applicability concerns. Thus, studies with a high risk of bias or applicability concerns were excluded from the quantitative analysis.

### 3.5. Fracture Patterns

In this section, we present the results for comparison of the diagnostic accuracy of complete and incomplete VRFs. Overall, the meta-analyses included 4863 CBCT images in five studies that directly compared the fracture patterns [8, 68, 85, 87, 100], 10 studies that assessed only complete VRFs [16, 22, 42, 43, 60, 61, 63, 70, 78, 81], and nine studies that assessed only incomplete VRFs [12, 18–20, 35, 96, 97, 102, 110]. There was a statistically significant (Chi-square = 12.98; *p*=0.0015) difference in the diagnostic accuracy according to the fracture pattern. It was observed that complete VRFs presented a higher sensitivity (SSe = 0.749; 95% CI, 0.675–0.811) than the incomplete pattern (SSe = 0.522; 95% CI, 0.424–0.618) (Chi-square = 12.95; *p*=0.0003; *I*^2^ = 86.0%). There was no significant difference (Chi-square = 1.51; *p*=0.2189) regarding the specificity of complete (SSp = 0.842; 95% CI, 0.792–0.882) or incomplete VRFs (SSp = 0.786; 95% CI, 0.716–0.843; *I*^2^ = 79.2%).

**Figure 4:**
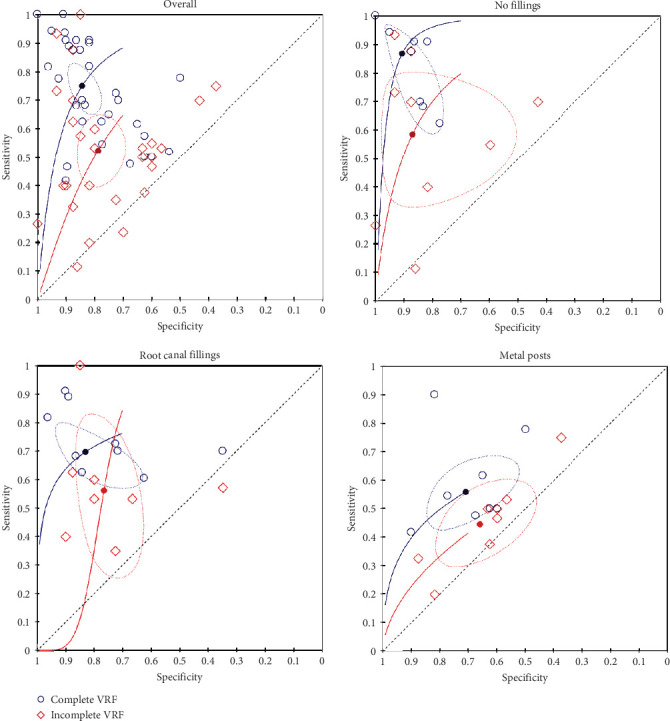
SROC plots of the meta-analyses for the overall comparison between complete and incomplete VRFs and in root canals with no fillings, gutta-percha, and posts.

Next, for a sensitivity analysis, the meta-analyses were conducted according to the root canal conditions. For root canals with no filling (i.e., empty canals), we performed a meta-analysis that included 1578 CBCT images, in three studies with direct comparisons between complete and incomplete VRFs [8, 85, 100], five studies that assessed only complete VRFs [16, 42, 43, 63, 78], and six studies that assessed only incomplete VRFs [12, 18, 19, 35, 96, 110]. There was a statistically significant (Chi-square = 18.26; *p*=0.0026) difference in the diagnostic accuracy according to the fracture pattern. The sensitivity of complete VRFs (SSe = 0.867; 95% CI, 0.721–0.942) was significantly higher (Chi-square = 18.26; *p*=0.0011) compared to the incomplete fracture pattern (SSe = 0.585; 95% CI, 0.367–0.775; *I*^2^ = 89.2%). The specificity did not differ (Chi-square = 0.3254; *p*=0.5684) between complete (SSp = 0.906; 95% CI, 0.831–0.949) and incomplete (SSp = 0.869; 95% CI, 0.714–0.946; *I*^2^ = 83.1%) VRFs (Figure 4).

Regarding teeth with root canal filling, the meta-analysis included the 1409 CBCT images, in three studies with direct comparisons [85, 87, 100], six studies that assessed only complete VRFs [16, 22, 42, 43, 78, 81], and five studies that assessed only incomplete VRFs [12, 20, 35, 96, 97]. The results revealed that there was no difference in the diagnostic accuracy of complete and incomplete VRFs (Chi-square = 3.62; *p*=0.163). Both sensitivity (SSe = 0.695; 95% CI, 0.616–0.764) and specificity (SSp = 0.829; 95% CI, 0.713–0.905) of the complete fractures were similar to the sensitivity (SSe = 0.561; 95% CI, 0.362–0.742; *I*^2^ = 81.7%) (Chi-square = 3.37; *p*=0.0664) and specificity (SSp = 0.764; 95% CI, 0.684–0.829; *I*^2^ = 78.7%) (Chi-square = 0.29; *p*=0.5902) of the incomplete pattern (Figure 4).

The meta-analysis for the root canals with metal posts included the 1088 CBCT images, in three studies with direct comparisons [68, 85, 100], five studies that evaluated only complete VRFs [16, 42, 43, 78, 81], and five studies that evaluated only incomplete VRFs [12, 20, 35, 96, 102]. There were no significant differences in the diagnostic accuracy between the two fracture patterns (Chi-square = 8.41; *p*=0.135), in terms of sensitivity (SSe = 0.588; 95% CI, 0.476–0.637; and 0.444; 95% CI, 0.35–0.542; *I*^2^ = 55.2%; for complete and incomplete VRFs, respectively) (Chi-square = 3.48; *p*=0.062) or specificity (SSp = 0.709; 95% CI, 0.598–0.799; and 0.659; 95% CI, 0.54–0.76; *I*^2^ = 70.2%; for complete and incomplete VRFs, respectively) (Chi-square = 0.41; *p*=0.5232) (Figure 4).

It was not possible to assess the differences in diagnostic accuracy between complete and incomplete fractures in root canals with other intracanal materials, such as fiberglass posts and bioceramic- and zirconium-based root canal fillings, due to the lack of primary studies.

### 3.6. CBCT Scan Voxel Size

The investigation of the influence of the spatial resolution in the diagnostic accuracy of VRFs was performed with different thresholds for the differentiation between high and low resolutions, using the voxel size as parameter. The meta-analyses were divided according to the presence of intracanal materials (Table 1).

### 3.7. Threshold for High Resolution: 0.2 mm

The voxel size of 0.2 mm was set as the threshold for high resolution at the initial meta-analysis; thus, any voxel size below this value was considered “low resolution.” The meta-analysis of root canals with no filling included 2284 CBCT images, in two studies with low resolutions [42, 63], and 21 studies with high resolutions [8, 12, 14, 16–19, 21, 35, 42, 43, 47, 52, 53, 63, 78, 85, 96, 100, 107, 110]. There was no significant difference between high (SSe = 0.768; 95% CI, 0.66–0.85; SSp = 0.832; 95% CI, 0.772–0.879) and low resolutions (SSe = 0.781; 95% CI, 0.415–0.947; *I*^2^ = 79%; SSp = 0.883; 95% CI, 0.722–0.956; *I*^2^ = 76.6%) in the diagnostic sensitivity (Chi-square = 0.008; *p*=0.9286) and specificity (Chi-square = 0.4757; *p*=0.5088).

Similarly, there was no difference in root canals obturated with gutta-percha regarding the use of high (SSe = 0.715; 95% CI, 0.634–0.785; SSp = 0.797; 95% CI, 0.736–0.846) or low resolutions (SSe = 0.641; 95% CI, 0.474–0.78; *I*^2^ = 80.3%; SSp = 0.919; 95% CI, 0.692–0.983; *I*^2^ = 82.3%) in terms of sensitivity (Chi-square = 0.2711; *p*=0.6026) and specificity (Chi-square = 2.556; *p*=0.1099). The meta-analysis included 2321 CBCT images, in three studies with low resolutions [22, 42, 81], and 21 studies with high resolutions [12, 14, 16, 20–22, 35, 42, 43, 47, 53, 75, 77, 78, 81, 85, 87, 96, 97, 100, 107].

**Figure 5:**
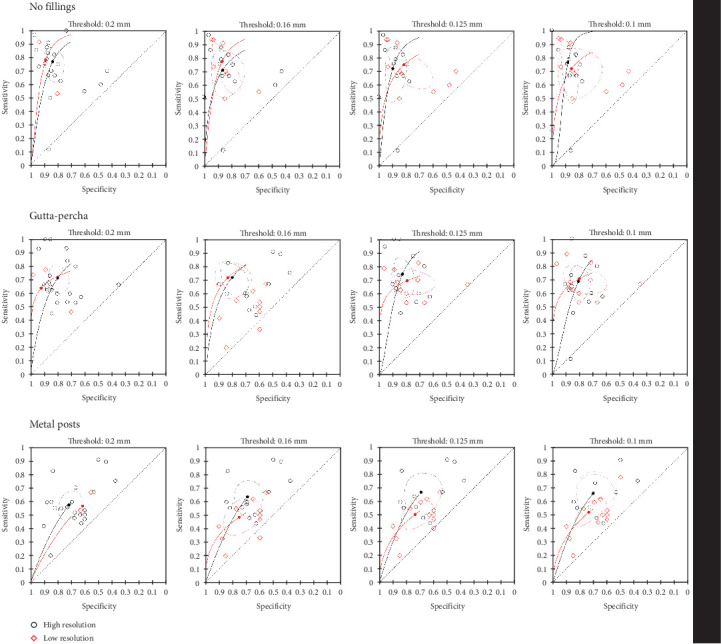
SROC plots of the meta-analyses for the comparisons between high and low spatial resolution considering different thresholds of voxel size, in root canals with no fillings, gutta-percha, and metal posts.

The meta-analysis of root canals with metal posts included 2052 CBCT images, in two studies with low resolutions [42, 81], and 21 studies with high resolutions [12, 14, 16, 17, 20, 21, 35, 42, 43, 47, 53, 68, 72, 78, 81, 85, 96, 98, 100, 102, 109]. There were also no significant differences in the sensitivity (Chi-square = 0.0143; *p*=0.9048) and specificity (Chi-square = 0.8098; *p*=0.3682) between high (SSe = 0.572; 95% CI, 0.492–0.65; SSp = 0.716; 95% CI, 0.648–0.774) and low (SSe = 0.564; 95% CI, 0.407–0.709; *I*^2^ = 76.3%; SSp = 0.615; 95% CI, 0.456–0.753; *I*^2^ = 72.4%) resolutions for the diagnostic accuracy of VRFs (Figure 5).

### 3.8. Threshold for High Resolution: 0.16 mm

When using the voxel size of 0.16 mm as the threshold for high spatial resolution, the meta-analysis of 2284 CBCT images of teeth with no fillings included eight studies with low resolution [12, 16, 17, 19, 42, 43, 63, 96], and 14 studies with high resolution [8, 14, 18, 21, 35, 47, 52, 53, 63, 78, 85, 100, 107, 110]. There was no significant difference between high (SSe = 0.708; 95% CI, 0.573–0.814; SSp = 0.865; 95% CI, 0.764–0.927) and low resolution (SSe = 0.772; 95% CI, 0.635–0.869; *I*^2^ = 78.2%; SSp = 0.868; 95% CI, 0.793–0.918; *I*^2^ = 83.3%) in sensitivity (Chi-square = 0.6131; *p*=0.4336) and specificity values (Chi-square = 0.0459; *p*=0.8304).

The meta-analysis of root canals obturated with gutta-percha included 2321 CBCT images in eight studies with low resolutions [12, 16, 22, 42, 43, 77, 81, 96], and 16 studies with high resolutions [14, 20–22, 35, 47, 53, 75, 77, 78, 81, 85, 87, 97, 100, 107]. There was also no significant difference between high (SSe = 0.716; 95% CI, 0.622–0.794; SSp = 0.803; 95% CI, 0.724–0.864) and low resolutions (SSe = 0.715; 95% CI, 0.619–0.795; *I*^2^ = 76.2%; SSp = 0.833; 95% CI, 0.731–0.902; *I*^2^ = 82%) in sensitivity (Chi-square = 0.0014; *p*=0.9704) and specificity values (Chi-square = 0.2756; *p*=0.5996).

Regarding the presence of metal posts, the meta-analysis included 2052 CBCT images in 10 studies with low resolutions [12, 16, 17, 42, 43, 68, 81, 96, 98, 102], and 12 studies with high resolutions [14, 20, 21, 35, 47, 53, 72, 78, 81, 85, 98, 100, 109]. It was shown that high resolution CBCT images presented higher sensitivity (SSe = 0.632; 95% CI, 0.539–0.716) (Chi-square = 6.408; *p*=0.0114) for the detection of VRFs, compared to low resolution (SSe = 0.482; 95% CI, 0.394–0.571; *I*^2^ = 76.5%). There was no difference between high (SSp = 0.689; 95% CI, 0.611–0.758) and low resolution (SSp = 0.749; 95% CI, 0.644–0.832; *I*^2^ = 72.5%) in the specificity values (Chi-square = 0.8946; *p*=0.3442) (Figure 5).

### 3.9. Threshold for High Resolution: 0.125 mm

The meta-analyses considering the voxel size of 0.125 mm as the threshold for high spatial resolution revealed that the diagnostic accuracy of teeth with no fillings did not differ between high (SSe = 0.72; 95% CI, 0.562–0.837; SSp = 0.896; 95% CI, 0.834–0.936) and low resolutions (SSe = 0.749; 95% CI, 0.635–0.836; *I*^2^ = 78.2%; SSp = 0.813; 95% CI, 0.683–0.897; *I*^2^ = 83.3%), in either sensitivity (Chi-square = 0.2135; *p*=0.644) or specificity (Chi-square = 2.7576; *p*=0.0968). The meta-analysis included 2284 CBCT images in 10 studies with low resolutions [12, 16, 18, 19, 36, 42, 43, 52, 63, 96], and 12 studies with high resolutions [8, 14, 21, 35, 47, 53, 63, 78, 85, 100, 107, 110].

Gutta-percha filled canals also presented no difference in a meta-analysis with 2321 CBCT images in 12 studies with high resolution [12, 16, 20, 22, 42, 43, 77, 81, 87, 96, 100, 107] (SSe = 0.742; 95% CI, 0.626–0.831; SSp = 0.827; 95% CI, 0.768–0.874), and 12 studies with low resolution [14, 21, 22, 35, 47, 53, 75, 77, 78, 81, 85, 97] (SSe = 0.694; 95% CI, 0.627–0.753; *I*^2^ = 75.4%; SSp = 0.792; 95% CI, 0.666–0.88; *I*^2^ = 82%), for both sensitivity (Chi-square = 0.3833; *p*=0.5358) and specificity (Chi-square = 0.5601; *p*=0.4542).

The meta-analysis of root canals with metal posts included 2052 CBCT images in 12 studies with low resolution [12, 16, 17, 20, 42, 43, 68, 72, 81, 96, 98, 102] (SSe = 0.502; 95% CI, 0.425–0.578; SSp = 0.735; 95% CI, 0.649–0.806), and 11 studies with high resolution [14, 21, 35, 47, 53, 78, 81, 85, 98, 100, 109] (SSe = 0.665; 95% CI, 0.551–0.762; *I*^2^ = 76.7%; SSp = 0.692; 95% CI, 0.593–0.776; *I*^2^ = 72.9%). It was observed a higher sensitivity for high-resolution CBCT scans (Chi-square = 6.9488; *p*=0.0084), compared to low resolution. There was no difference regarding the specificity (Chi-square = 0.3108; *p*=0.5772) (Figure 5).

### 3.10. Threshold for High Resolution: 0.1 mm

Using the voxel size of 0.1 mm as the threshold, the meta-analysis of root canals with no filling included 2284 CBCT images in 11 studies with low resolutions [8, 12, 16–19, 42, 43, 52, 63, 96] (SSe = 0.717; 95% CI, 0.567–0.83; SSp = 0.855; 95% CI, 0.718–0.932), and 11 studies with high resolutions [8, 14, 21, 35, 47, 53, 78, 85, 100, 107, 110] (SSe = 0.763; 95% CI, 0.569–0.888; *I*^2^ = 86.6%; SSp = 0.881; 95% CI, 0.832–0.917; *I*^2^ = 83.2%), and no difference was observed regarding the diagnostic accuracy of VRF, in terms of sensitivity (Chi-square = 0.0917; *p*=0.7621) and specificity (Chi-square = 0.6498; *p*=0.4202).

Gutta-percha-filled root canals also presented no difference regarding the sensitivity (Chi-square = 0.1955; *p*=0.6584) and specificity (Chi-square = 0.0635; *p*=0.8011), in a meta-analysis with 2321 CBCT images in 10 studies with low resolution [12, 16, 20, 22, 42, 43, 77, 81, 87, 96] (SSe = 0.707; 95% CI, 0.633–0.771; SSp = 0.795; 95% CI, 0.67–0.881) and 12 studies with high resolution [14, 21, 35, 47, 53, 75, 77, 78, 85, 97, 100, 107] (SSe = 0.686; 95% CI, 0.588–0.771; *I*^2^ = 75.1%; SSp = 0.802; 95% CI, 0.746–0.848; *I*^2^ = 83.6%).

The meta-analysis of root canals with metal posts included 2052 CBCT images in 12 studies with low resolutions [12, 16, 17, 20, 42, 43, 68, 72, 81, 96, 98, 102] (SSe = 0.517; 95% CI, 0.445–0.588; SSp = 0.734; 95% CI, 0.649–0.805), and 10 studies with high resolutions [14, 21, 35, 47, 53, 78, 85, 98, 100, 109] (SSe = 0.654; 95% CI, 0.539–0.753; *I*^2^ = 77.6%; SSp = 0.702; 95% CI, 0.605–0.785; *I*^2^ = 73.6%). It was observed a higher sensitivity for high-resolution CBCT scans (Chi-square = 5.0178; *p*=0.0251), compared to low resolution. There was no difference regarding the specificity (Chi-square = 0.1384; *p*=0.7099) (Figure 5).

It was not possible to assess the influence of the spatial resolution on the detection of VRFs in teeth with other intracanal materials due to the lack of primary studies.

### 3.11. Presence of Intracanal Materials

The effect of intracanal materials on the diagnostic accuracy of VRFs was assessed using empty root canals as the comparator for the meta-analyses of each material. Sensitivity analyses were conducted, when possible, to assess the influence of the intracanal materials, within each type of fracture (i.e., complete or incomplete), on the diagnostic accuracy of VRF.

### 3.12. No Filling × Gutta-Percha

**Figure 6:**
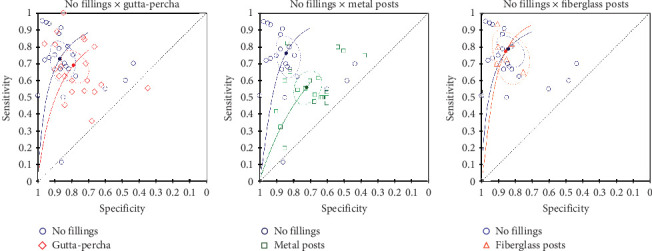
SROC plots of the meta-analyses for the comparisons among different intracanal materials, using root canal with no fillings as comparator.

The meta-analysis that compared empty root canals and root canals obturated with gutta-percha included 4119 CBCT images in 14 studies that compared both intracanal conditions [12, 14, 16, 21, 35, 42, 43, 47, 78, 79, 85, 96, 100, 107], eight studies that assessed empty root canals [8, 17–19, 52, 58, 63, 110], and seven studies with gutta-percha-filled root canals [20, 22, 75, 77, 81, 87, 97]. There was no significant difference between the diagnostic accuracy of teeth with empty canals (SSe = 0.726; 95% CI, 0.627–0.807; SSp = 0.87; 95% CI, 0.804–0.916) or with gutta-percha-based root canal fillings (SSe = 0.688; 95% CI, 0.611–0.756; *I*^2^ = 81.7%; SSp = 0.789; 95% CI, 0.724–0.842; *I*^2^ = 84%) in terms of sensitivity (Chi-square = 0.2526; *p*=0.6152) and specificity (Chi-square = 3.0317; *p*=0.0816) (Figure 6).

However, the sensitivity analysis demonstrated that in the presence of complete VRFs [8, 22, 42, 43, 63, 78, 81, 85, 87, 100], there was a significant difference in the diagnostic accuracy of empty root canals (SSe = 0.862; 95% CI, 0.726–0.936; SSp = 0.899; 95% CI, 0.83–0.941) and root-filled canals (SSe = 0.71; 95% CI, 0.636–0.775; *I*^2^ = 81.8%; SSp = 0.815; 95% CI, 0.676–0.903; *I*^2^ = 84.6%), in the sensitivity (Chi-square = 3.1803; *p*=0.03453). There was no difference in the specificity (Chi-square = 2.1005; *p*=0.1472). In contrast, teeth with incomplete VRFs [8, 12, 18–20, 35, 58, 85, 87, 96, 97, 100, 110] presented no difference in the diagnostic accuracy of empty root canals (SSe = 0.655; 95% CI, 0.419–0.833; SSp = 0.892; 95% CI, 0.758–0.956) or root-filled canals (SSe = 0.594; 95% CI, 0.412–0.753; *I*^2^ = 84.8%; SSp = 0.751; 95% CI, 0.622–0.846; *I*^2^ = 82.4%) in sensitivity (Chi-square = 0.0533; *p*=0.8174) and specificity (Chi-square = 2.0482; *p*=0.1524) values.

The majority of the studies did not use endodontic sealers and gutta-percha cones [12, 16, 35, 42, 43, 47, 78, 79, 87, 96, 100, 107] to avoid the penetration of the filling material within the fracture line. Other studies assessed root canals filled with gutta-percha and epoxy resin-based sealers [21, 22, 77, 97], zinc oxide-eugenol-based sealer [77, 81], and calcium silicate-based sealer [75]. Two studies failed to describe whether an endodontic sealer was used or not [14, 20].

### 3.13. No Filling × Metal Posts

The meta-analysis comparing empty root canals and root canals with metal posts included 3537 teeth in 13 studies that compared both situations [12, 14, 16, 17, 21, 35, 42, 43, 47, 78, 85, 96, 100], nine studies that assessed empty root canals [8, 18, 19, 52, 58, 63, 79, 107, 110], and seven studies with metal posts [20, 68, 72, 81, 98, 102, 109]. It was found that teeth with metal posts present a lower diagnostic accuracy (SSe = 0.56; 95% CI, 0.485–0.632; SSp = 0.719; 95% CI, 0.653–0.777) than teeth with no root canal fillings (SSe = 0.763; 95% CI, 0.658–0.844; *I*^2^ = 85.5%; SSp = 0.841; 95% CI, 0.781–0.887; *I*^2^ = 77.3%), in terms of sensitivity (Chi-square = 8.0378; *p*=0.0046) and specificity (Chi-square = 7.6206; *p*=0.0058) (Figure 6).

These findings were also confirmed by the sensitivity analysis according to the fracture patterns, and the results were similar for both complete and incomplete VRFs, with a significant difference between empty root canals and root canals with metal posts. In teeth with complete VRFs [8, 16, 42, 43, 63, 68, 78, 81, 85, 100], the empty root canals (SSe = 0.867; 95% CI, 0.721–0.942; SSp = 0.906; 95% CI, 0.831–0.949) presented a higher diagnostic accuracy than teeth with metal posts (SSe = 0.558; 95% CI, 0.476–0.637; *I*^2^ = 89.8%; SSp = 0.709; 95% CI, 0.598–0.799; *I*^2^ = 82.4%), in both sensitivity (Chi-square = 8.1138; *p*=0.0044) and specificity (Chi-square = 7.1795; *p*=0.0074).

Similarly, in teeth with incomplete VRFs [8, 12, 18–20, 35, 58, 68, 85, 96, 100, 102, 110], the empty root canals (SSe = 0.655; 95% CI, 0.419–0.833; SSp = 0.892; 95% CI, 0.748–0.965) also presented higher diagnostic accuracy than teeth with metal posts (SSe = 0.439; 95% CI, 0.344–0.539; *I*^2^ = 83.3%; SSp = 0.665; 95% CI, 0.544–0.767; *I*^2^ = 83%), in both sensitivity (Chi-square = 3.8004; *p*=0.0397) and specificity (Chi-square = 5.2118; *p*=0.0224).

The included studies used different types of metallic posts, such as gold alloys [16, 42, 43, 47, 100], nickel–chromium [14, 21, 47, 81, 98], cobalt–chromium [68, 78, 85, 96, 102, 109], and silver–palladium [72]. Some studies failed to report the type of metal posts that were tested [12, 17, 20, 35].

### 3.14. No Filling × Fiberglass Posts

The diagnostic accuracy of teeth with fiberglass posts (SSe = 0.773; 95% CI, 0.677–0.847; SSp = 0.855; 95% CI, 0.788–0.903) did not differ from teeth with no root canal fillings (SSe = 0.786; 95% CI, 0.709–0.847; *I*^2^ = 76.5%; SSp = 0.841; 95% CI, 0.772–0.891; *I*^2^ = 73.9%), in terms of either sensitivity or specificity (Chi-square = 0.5218; *p*=0.7704). The meta-analysis included 2212 teeth in five studies that compared both clinical situations [16, 47, 78, 85, 100], 15 studies with empty root canals [8, 12, 14, 17–19, 21, 35, 42, 43, 52, 58, 63, 79, 96], and 1 study with fiberglass posts [70] (Figure 6).

In the sensitivity analysis, these findings were confirmed. There was no significant difference in the diagnostic accuracy of complete VRFs [8, 16, 42, 43, 63, 70, 78, 85, 100], between teeth with fiberglass posts (SSe = 0.855; 95% CI, 0.72–0.931; SSp = 0.855; 95% CI, 0.767–0.913) and empty root canals (SSe = 0.867; 95% CI, 0.721–0.942; *I*^2^ = 82.8%; SSp = 0.906; 95% CI, 0.831–0.949; *I*^2^ = 65.4%), in both sensitivity (Chi-square = 0.0006; *p*=0.9797) and specificity (Chi-square = 0.6209; *p*=0.4307). Similarly, there was no difference in the diagnostic accuracy of incomplete VRFs [8, 12, 18, 19, 35, 58, 85, 96, 100], between teeth with fiberglass posts (SSe = 0.529; 95% CI, 0.412–0.642; SSp = 0.873; 95% CI, 0.774–0.933) and empty root canals (SSe = 0.717; 95% CI, 0.523–0.854; *I*^2^ = 86.6%; SSp = 0.899; 95% CI, 0.743–0.965; *I*^2^ = 84.4%), in both sensitivity (Chi-square = 1.228; *p*=0.2678) and specificity (Chi-square = 0.0016; *p*=0.9681).

It was not possible to assess the influence of other intracanal materials on the diagnostic accuracy of VRFs due to the limited number of primary studies.

### 3.15. Image Enhancement Filters

Three studies evaluated the use of sharpness filters in the i-CAT vision software (Imaging Sciences International, Hatfield, PA, USA). The filters were sharpen [15, 17, 19], hard [15], sharpen mild [17, 19], sharpen 3 × 3 [17, 19], s9 [17, 19], smooth [17], smooth 3 × 3 [17], sharpen super mild [17], angio sharpen medium 5 × 5 [17], angio sharpen high 5 × 5 [17], and shadow 3 × 3 [17]. The Adaptive Image Noise Optimizer filter [46] (AINO, Planmeca, Helsinki, Finland), and the sharpness filters in the OnDemand 3D software (Cybermed, Irvine, CA, USA) [109], were assessed by one study each. The use of enhancement filters in CBCT images demonstrated no influence on the diagnosis of VRFs in teeth with no fillings [17, 19], root canal filling [15, 46], or metal posts [15, 17, 109]. Therefore, the evidence suggests that their use is not justified.

The use of artifact reduction filters was assessed by different studies, in teeth with no filling [14, 65, 100], root canal filling [14, 49, 65, 100], fiberglass posts [100], and metal posts [14, 65, 100]. The filters were: MATLAB artifact removal software [49] (MathWorks Inc, Natick, MA, USA); and Blooming Artifact Reduction filter [14, 65, 100] (BAR, e-Vol DX, CDT software, Bauru, SP, Brazil). The results were not consistent, and although some level of improvement in diagnostic accuracy was observed when using MATLAB artifact removal software [49] and BAR filter [14], it was dependent on the CBCT device that was used.

### 3.16. MAR Algorithms

Several studies investigated the diagnostic accuracy of CBCT scans acquired with MAR algorithms using the standard CBCT scans as comparators [13, 16, 18, 20–22, 49, 56, 68, 74, 76, 86, 93, 102–104, 106, 107, 109, 111, 114]. The algorithms evaluated were: ProMax MAR algorithm [13, 20, 49, 56, 76, 86, 93] (Planmeca, Helsinki, Finland); Master 3D MAR algorithm [13] (Vatech, Hwaseong, South Korea); Picasso Trio MAR algorithm [68, 102] (Vatech, Hwaseong, South Korea); Pax-i3D MAR algorithm [111] (Vatech Co., Ltd., Gyeonggi-do, South Korea); EasyDent4 MAR algorithm [16] (E-WOO, Giheung-gu, South Korea); OP300 MAR algorithm [18, 21, 74, 103, 106, 109] (Instrumentarium, Tuusula, Finland); Cranex 3D MAR algorithm [49] (Soredex, Tuusula, Finland); SMAR algorithm [114] (Scanora 3D, Soredex, Tuusula, Finland); i-CAT MAR algorithm [22] (Imaging Sciences International, Hatfield, PA, USA); 9600 3D MAR algorithm (Carestream, Rochester, NY, USA); and Midmark EIOS MAR postprocessing algorithm [107] (Midmark, Dayton, USA). The algorithms were applied in teeth with no fillings [16, 20, 21, 106, 107], root canal fillings [13, 16, 20–22, 49, 56, 106, 107, 111], metal posts [16, 20, 21, 68, 86, 93, 102, 103, 109, 111, 114], and fiberglass posts [16, 20]. Four studies evaluated the use of MAR algorithms for dental implants adjacent to the fractured teeth [74, 76, 104, 107], and one study assessed teeth with metal posts adjacent to the fractured teeth [18].

The results were very distinct among the included studies. Seven studies found no difference in the diagnostic accuracy of VRFs with or without MAR algorithms [16, 22, 49, 86, 93, 102, 114], while five studies reported some level of improvement in diagnostic accuracy when the algorithm was used [20, 56, 103, 106, 109, 111]. However, in some studies, the diagnosis improvement was influenced by the kilovoltage [56], intensity of MAR algorithm [56] (i.e., low, medium, or high), the type of intracanal material [103, 106], and the combined use of MAR algorithm and sharpness filters [109]. Additionally, in four studies the, use of MAR algorithms demonstrated a negative impact on the diagnostic accuracy of VRFs, compared to CBCT scans without algorithms [13, 21, 68, 107].

### 3.17. CBCT Device

The influence of the CBCT device on the diagnostic accuracy of VRF was evaluated by several studies [8, 13, 14, 33, 36–38, 49, 51, 53, 61, 70, 77, 89, 98]. The CBCT devices evaluated were: ProMax 3D (Planmeca) [13, 33, 49, 70, 110, 113]; Master 3D (Vatech) [13, 33]; 3D Accuitomo-XYZ (J. Morita, Kyoto, Japan) [61]; 3D Accuitomo 170 (J. Morita) [8, 37, 51]; i-CAT (Imaging Sciences International) [8, 36, 61, 77, 98]; PreXion 3D (Teracom, San Mateo, CA, USA) [14, 77]; OP300 (Instrumentarium) [14, 53, 108]; CS 9300 (Carestream) [70, 105]; CS 9000 3D (Carestream) [14, 53]; NewTom GO (Quantitative Radiology, Verona, Italy) [70]; NewTom 3G (Quantitative Radiology) [37, 38, 49, 51, 61]; NewTom VGi (Quantitative Radiology) [89, 113]; Scanora 3D (Soredex) [36, 61, 89, 113]; Orthophos XG (Sirona Dental System, Bensheim, Germany) [77]; Galileos 3D (Sirona Dental System) [61]; Iluma Ultra (Imtec Imaging, Ardmore, OK, USA) [38]; Cranex 3D (Soredex) [49, 105]; 3D eXam (Kavo Dental, Biberach, Germany) [113]; Eagle 3D V-Beam (Dabi Atlante, Ribeirão Preto, Brazil) [98]; Midmark EIOS [107] (Midmark, Dayton, USA); Veraviewspocs 3D R100 [65] (J Morita, Kyoto, Japan); and Veraview X800 [65, 108] (J Morita, Kyoto, Japan).

### 3.18. FOV Size

The influence of the FOV size was assessed by five studies in teeth with root canal filling and metal posts. Small FOVs assessed by the studies included: 4 × 4 [51, 88]; 5 × 5 [72]; 6 × 6 [45, 51]; and 10 × 7.5 cm [90]; while large FOVs included 8 × 8 [72]; 10 × 10 [88]; 13 × 14 [90]; 15 × 15 [51]; 18 × 16 [45]; and 22 × 22 cm [51]. The voxel size varied from 0.08 to 0.3 mm. In all studies, it was observed that small-volume CBCT scans presented higher diagnostic accuracy for VRFs than CBCT scans with large FOVs.

### 3.19. Position Within FOV

Three studies assessed the influence of the position of the suspected tooth within the FOV, on the diagnostic accuracy of VRF. The studies evaluated teeth with no filling [20], root canal filling [20], metal posts [7, 20, 72], and fiberglass posts [20]. The FOV sizes varied from 5 × 5 to 15 × 15 cm, whilst the voxel size varied between 0.16 and 0.2 mm. It was consensus among all studies that the central positioning of the suspected tooth increased diagnostic accuracy, compared to teeth positioned in the peripheral area of the FOV.

### 3.20. Tube Current (Milliamperage)

Eight studies evaluated the association of the CBCT tube current and the diagnostic accuracy of VRFs [18, 41, 47, 72, 74, 78, 90, 105], in teeth with no filling [18, 47, 74, 78], root canal filling [41, 47, 78, 105], metal posts [47, 72, 78, 90, 105], and fiberglass posts [47, 78, 105]. The tube currents of 2, 4, 5, 6, 6.3, 7, 8, 10, 12, and 13 mA were assessed. In the majority of studies, there was no influence of lower or higher tube currents in the diagnostic task [18, 47, 72, 74, 78, 92, 105]. Conversely, one study found the increase in tube current improved diagnostic accuracy [41], while another found better diagnostic performance with lower tube current [90].

### 3.21. Tube Voltage

Six studies assessed the influence of the CBCT tube voltage on the diagnostic accuracy of VRFs [31, 41, 47, 56, 76, 102], in teeth with no filling [47, 76], root canal filling [41, 47], fiberglass posts [47], and metal posts [31, 47]. The tested tube voltage used for image acquisition included 60, 70, 74, 80, 86, 90, 92, and 99 kVp. In four studies [47, 56, 76, 102], the variations in tube voltage did not alter the diagnostic accuracy. In contrast, in one study 60 kVp performed better than 85 kVp [41], and in another study, 80 kVp performed better than 92 kVp [31].

### 3.22. Metallic Objects Inside the FOV

Seven studies evaluated the influence of the presence of metallic objects (i.e., metal posts, dental implants) adjacent to the investigation tooth [18, 70, 71, 74, 76, 104, 107]. The voxel sizes varied from 0.078 to 0.15 mm. The included studies used titanium implants [70, 71, 107], nickel–titanium implants [104], zirconium-oxide implants [74, 76], nickel–chromium metal posts [18], and cobalt–chromium metal posts [70, 71], to generate artifacts. The results are somewhat inconsistent. Five studies reported a decreased diagnostic accuracy in the presence of metallic objects [18, 74, 76, 104, 107], and two studies found no difference regarding the presence of such objects [70, 71].

Meta-analyses could not be performed to evaluate the influence of image enhancement filters, MAR algorithms, CBCT devices, FOV size, positioning within the FOV, tube current, or tube voltage on the diagnostic accuracy of VRFs, primarily due to the limited number of primary studies and high methodological heterogeneity. The included studies varied widely in terms of comparisons, intracanal materials, metallic objects, and acquisition parameters—such as FOV and voxel size, tube current, and tube voltage—making it difficult to extrapolate their findings or draw consistent conclusions across studies. Also, it was not possible to assess the influence of other possible factors in the diagnostic accuracy of VRFs using CBCT scans, as only one study was available. These factors are bioceramic root-filling material [67], zirconium-based root-filling material [75], and DICOM viewer software [43].

### 3.23. Quality of Evidence

The assessment of the quality of evidence based on the GRADE approach revealed a “very low” certainty of evidence for the meta-analyses that evaluated the influence of the fracture type, voxel size, and intracanal materials. The downgrade was applied at the domains of indirectness, due to the in vitro nature of the primary studies, and inconsistency, due to the high statistical heterogeneity among the included studies. Further details regarding the GRADE assessments are available in the summary-of-findings table (Table 2).

## 4. Discussion

Diagnosing VRFs is difficult in dental practice, as it is identified in most clinical situations by a combination of signs, symptoms, and radiographic findings. There is no consensus on the diagnostic accuracy of CBCT images for VRFs, and many factors might be involved. Studies have evaluated the influence of the intracanal materials [12, 14–17, 20, 21, 29, 30, 35, 36, 39, 42–45, 47, 48, 50, 53, 59, 65, 67, 75, 78–81, 83–85, 88, 92, 96, 99, 107–111], fracture pattern [8, 34, 40, 68, 85, 87, 112], image acquisition parameters [12, 13, 15, 18, 22, 31–33, 38, 41, 42, 45–47, 50, 51, 59, 63, 65, 66, 69, 72, 74, 76, 78, 81, 88, 90, 97, 98, 107–110], and other factors. Current evidence regarding in vivo detection of VRFs is very limited [116], and it would be impossible to identify the factors involved in diagnostic accuracy using only these studies. Hence, since there is a high volume of data in published articles, the present systematic review focused on in vitro studies that evaluated the diagnosis of VRF by CBCT imaging. We intended to screen the main factors that might optimize CBCT imaging for the diagnosis of VRFs and guide further laboratory and clinical investigations. Meta-analyses were performed to assess the influence of each factor on sensitivity and specificity values in the diagnosis of VRFs. The quality of evidence was also assessed for each factor.

The effect of intracanal materials on the diagnostic accuracy of VRFs was assessed, using nonendodontically treated teeth for comparison. It was observed that the presence of root canal filling caused a reduction in sensitivity and specificity values only in teeth with complete VRFs. This may be explained by the fracture width of complete and incomplete VRFs. The mean gap between fragments of incomplete VRFs is much lower than complete ones [8, 87]. Hence, the effects of imaging artifacts caused by gutta-percha are much more prejudicial when assessing complete VRFs, which would be visible in the absence of artifacts. In contrast, incipient fractures [85, 87, 112], with minimal separation between fragments [4, 117], are not easily detected by CBCT images, as the spatial resolution might not allow the reconstruction of the fracture lines, regardless of the presence of root canal filling. Conversely, it was observed that the presence of metal posts caused a reduction in both sensitivity and specificity values in teeth with either complete or incomplete VRFs. These findings were expected because the beam-hardening artifacts are more prominent in the presence of materials containing chemical elements with higher atomic numbers [10]. The high radiodensity of metal posts severely affects image quality [45, 89]. Our findings corroborate with Dias et al. [118], which recently analyzed in vivo VRFs, and found that intracanal posts limited the diagnostic performance of CBCT images [118].

Also, regarding imaging artifacts, the influence of metallic objects inside the FOV on the diagnosis of VRFs has been investigated [18, 70, 71, 74, 76, 107]. Nickel–chromium [18] and cobalt–chromium [70, 71] metal posts, and dental implants composed mainly of titanium [70, 71, 107] or zirconium [74, 76] were analyzed. Three studies reported that the presence of metallic objects impaired the diagnostic performance of CBCT images [18, 74, 76]. Specificity is significantly lower in the presence of zirconium implants [74, 76]. The artifacts formed in adjacent areas may mimic fracture lines, generating FP diagnoses. In opposition, three studies reported no influence of cobalt–chromium posts or titanium implants on the diagnosis of VRF in adjacent teeth [70, 71, 107]. All included studies used dry human mandibles as phantoms, and high-resolution CBCT images, with voxel sizes that ranged from 0.08 to 0.15 mm. Perhaps other factors also influenced the diagnostic task, such as fracture pattern, CBCT device, and tube current and voltage. The influence of metallic objects within the FOV should be further investigated.

The final quality of CBCT images is affected by several technical factors. Changes in the acquisition parameters have been thoroughly evaluated by several studies, in terms of tube current [18, 41, 47, 72, 74, 78, 90], tube voltage [31, 41, 47, 76], and FOV size [45, 51, 72, 88, 90], however, no consensus was found. Regarding the FOV size, it is consensus that small FOVs present superior diagnostic performance in comparison with the large ones [45, 51, 72, 88, 90]. Most studies found no discrepancies in the diagnostic accuracy of VRFs when varying the tube current in nonendodontically treated teeth [18, 47, 74, 78], root-filled teeth [47, 78], and teeth with metal [47, 72, 78], and fiberglass posts [47, 78]. In contrast, one study reported better diagnostic performance with increased tube current in root-filled teeth [41], and in another study, the diagnosis of teeth with metal posts was improved with a reduction in tube current [90]. Changes in tube voltage also provided controversial results. Two studies reported no difference in the detection of VRFs with varied tube voltage, regardless of the intracanal material [47, 76]. Conversely, other two studies reported that a reduction in tube voltage improved the diagnostic accuracy of VRFs, in root-filled teeth [41], and roots with metal posts [31]. Adjustments in tube current and voltage cause variations in the emitted radiation, as they modify the beam energy, penetrability, and the number of photons that reach the CBCT detector [119]. Bearing this in mind, variations in acquisition parameters are focused on reducing the effective radiation dose, following the ALARA—as low as reasonably achievable—principles [120, 121]. However, there is no reliable evidence that the effective dose of CBCT scans presents any biological risk, regarding the incidence of cancer or raising the mortality rates [120, 121]. Hence, further research efforts should focus solely on developing strategies and tools that may improve CBCT image quality.

The main factor that influences the spatial resolution of CBCT images is the voxel size. The meta-analyses that evaluated the influence of the voxel size were divided according to different thresholds set for high and low resolutions. According to our findings, a minimum voxel size of 0.16 mm should be considered to optimize VRF diagnosis in teeth with metal posts. However, it is important to clarify that only the sensitivity was improved with higher spatial resolutions. The specificity was unchanged with voxel-size variations. The summarized specificity for teeth with metal posts was 0.719 (95% CI, 0.653–0.777). A lower specificity is observed in the presence of FP diagnoses. FP rates are raised in teeth with metal posts due to the presence of artifacts that mimic the presence of fracture lines [15, 42, 50, 81, 98]. Since most teeth with VRF are extracted [4, 117], clinicians should be cautious when assessing VRFs in teeth with metal posts [10, 39, 87]. Direct visualization of the fracture line by surgery might be necessary to validate the diagnosis [6, 117]. Conversely, the diagnosis of VRFs in teeth filled with gutta-percha, or nonroot-filled teeth, does not seem to be influenced by the voxel size, according to our results. PradeepKumar et al. [116]. recently assessed the diagnostic accuracy of in vivo VRFs by meta-analysis for root-filled teeth. The meta-regression did not identify the voxel size as a possible confounding factor. Conversely, de Lima et al. [122]. Investigated the influence of CBCT acquisition parameters by meta-analysis of in vitro and observational studies. The authors concluded that smaller FOV and voxel sizes provided more accurate detection of VRFs.

Although voxel size does not influence the formation of beam-hardening artifacts, it is directly associated with the spatial resolution of CBCT images—smaller voxels result in higher resolution. When materials of different densities, such as a metal post and dentin, occupy the same voxel, a phenomenon known as the partial volume effect (PVE) may occur, leading to errors in image acquisition and potential underestimation of material volumes [10]. Reducing voxel size minimizes the impact of the PVE [123], which may help explain the improved diagnostic accuracy of high-resolution CBCT images in detecting VRFs, where the subtle fracture lines can be obscured by this effect. Additionally, improved spatial resolution reduces blooming artifacts—hyperdense halos surrounding high-density objects like metal posts—which cause an overestimation in the volume of the metal post and can interfere with the visualization of fracture lines [10, 100, 123].

Some CBCT units offer MAR algorithm options for image reconstruction. The use of MAR algorithms remains controversial. Most of the studies reported that there is no influence on the diagnostic accuracy of VRF when using these tools [16, 22, 49, 86, 93, 107, 109, 114]. In some studies, the MAR algorithms impaired diagnostic performance [13, 21, 68]. Since subtle VRFs present small hypodense lines, which are very similar to an artifact streak, the image enhancement provided by the MAR algorithms did not improve diagnostic accuracy [124]. The mechanism of MAR algorithms includes interpolation-based sinogram corrections, which replace the projection data of metal objects with surrounding data in the sinogram [16]. However, studies have reported incomplete artifact correction [125], secondary artifact formation [125], and loss of information surrounding high-density materials [13]. Conversely, two studies reported that MAR algorithms improved the diagnostic sensitivity of VRFs [20, 111]. However, the main improvement reported in these studies occurred in teeth without root canal filling. Hence, the applicability of MAR algorithms for the diagnosis of VRFs is, at minimum, questionable. It is important to consider that most of the included studies assessing AR tools presented a high or moderate risk of bias [13, 18, 20, 49, 76, 86, 93, 111, 114]. The main concerns regarding the risk of bias were the lack of a baseline evaluation to exclude previously fractured teeth, incomplete description of the reference standard test (i.e., direct visualization, magnification, and transillumination), or restriction of the reference standard test to the fractured teeth.

Enhancement filters also have been assessed to reduce the impact of imaging artifacts. Filters differ from MAR algorithms as they are applied to an acquired image and not during CBCT acquisition. The mechanism is based on increasing or decreasing image characteristics, such as sharpness and noise. It has been shown that applying enhancement filters in tomographic images did not improve the diagnostic accuracy of VRFs [15, 17, 19, 46, 109]. Alternatively, some enhancement filters use different mechanisms, based on enhancing gray-scale contrast, to reduce artifacts in CBCT images [14, 49, 65, 100]. Although it has been demonstrated that these filters improved image quality [126, 127], there is no change in the beam-hardening effects in CBCT acquisitions. The evidence regarding the diagnostic accuracy of CBCT images with artifact removal filters is uncertain. Saati et al. [49] evaluated the MATLAB artifact removal filter in root-filled teeth, using NewTom 3G, ProMax 3D, and Cranex 3D. The MATLAB filter improved the diagnostic accuracy when using the Cranex 3D images. However, the authors did not describe the type of fracture pattern that was evaluated and the voxel size provided by each CBCT unit. Similarly, Caetano et al. [14] also evaluated an artifact removal filter, e-vol DX BAR filter, in nonendodontically treated teeth, root-filled teeth, and metal posts. The BAR filter improved the overall accuracy of the 9000-3D device (from 62% to 74%). However, the accuracy was still lower than the OP300 and PreXion 3D units (86% and 96%, respectively), regardless of the application of the BAR filter (87% and 92%, respectively). de Lima Dias-Junior et al. [100] also observed that the BAR filter did not improve the diagnosis of complete and incomplete VRFs, in teeth with root canal fillings, fiberglass posts, or metal posts, using the PreXion 3D device. Hence, it seems that using different CBCT devices has more impact on the final image quality than the application of enhancement filters.

Regarding the diagnostic accuracy provided by different CBCT devices, several units have been compared [8, 13, 14, 33, 36–38, 49, 51, 53, 61, 70, 77, 89, 98]. It has been argued that the technology of CBCT detectors affects image quality in terms of spatial resolution, dynamic range, and contrast resolution [61]. Detectors based on image intensifier tube/charged coupled device combinations (IIT/CCD) presented inferior diagnostic performance compared to flat-panel detectors (FPD) in some studies [37, 61], while others found no difference [38, 49, 51]. It was not possible to perform a meta-analysis, because the studies present high methodological heterogeneity. There are important differences among the included studies in the fracture pattern, intracanal materials, and acquisition parameters, which directly impact the image quality, and diagnostic performance. Nevertheless, the majority of the included studies obtained CBCT images on FPD-based devices [7, 8, 12–22, 29–51, 53–55, 58–64, 66–78, 80–99, 112, 114, 115], and a few studies used ITT/CCD devices [37, 38, 49, 51, 52, 61, 79, 111].

Regarding the quality assessment of the included studies, most of the studies presented a high risk of bias. The main reason why several studies presented high risk of bias was the reference standard test adopted by them. Low risk of bias was recognized for studies that described that all specimens were analyzed before and after fracture induction, using at least one method to assist the direct visualization of the fracture lines: magnification of any type, transillumination, or application of stains [7, 8, 12, 14–19, 21, 22, 29–31, 34–36, 38, 40, 42–45, 47, 50, 52–54, 58–63, 66, 68, 70–72, 74–87, 89, 90, 92–95, 97, 112]. Unclear risk of bias was decided for studies that solely described the reference standard as direct visualization [20, 88, 96, 98]. However, several studies did not describe the reference standard test [13, 32, 33, 46, 73, 99], or did not evaluate all specimens with the test [37, 39, 41, 48, 49, 51, 67, 69, 74, 76, 80, 91, 93, 94, 111, 112, 114, 115], and thus, were considered as high risk of bias. The diagnostic accuracy of an index test is determined after establishing the presence or absence of the target condition in the sample with the reference standard test. As several tests may be available to detect the same condition, the authors should describe the methods used for both index and reference standard tests in sufficient detail to allow replication [128]. Differences in test protocols potentially affect the variability in accuracy measures among studies.

Also, a high risk of bias was attributed to several studies due to the lack of anthropomorphic model designs [7, 12, 30, 34, 36, 39, 40, 44, 45, 48–50, 59, 66, 80, 84, 86, 89, 90, 93, 94, 99, 111, 112, 114, 115], to simulate the clinical condition of a CBCT scan. Andraws Yalda et al. [129] recently investigated the influence of different phantom designs on the diagnosis of root fractures. The authors evaluated five models (i.e., acrylic resin block, human skull, skull covered in wax, skull immersed in water, and skull immersed in water with cervical vertebrae) and found no difference among the experimental designs. However, the authors did not compare the in vitro models with an in vivo assessment. In vivo, a CBCT scan is affected by scattering artifacts and image noise, due to the X-ray attenuation by the patient's soft and hard tissues. Acquiring images without any simulation of the clinical condition provides a level of contrast and a contrast-to-noise ratio that is impossible to achieve in any in vivo scenario. Hence, it is not plausible to assume that images taken from teeth in acrylic resin or gypsum blocks represent a valid simulation of real CBCT scans for the diagnosis of VRFs.

This study attempted to rigorously select the most appropriate in vitro studies regarding the simulation of clinical conditions (i.e., use of anthropomorphic model designs and fracture induction) for the quantitative analysis. However, it is important to address the limitations of this systematic review. The CBCT image acquired in laboratory settings achieves a quality that is unmanageable in a clinical setting. The X-ray beam attenuation differs markedly from in vivo image acquisitions due to the absence of soft and hard tissues, which can influence image noise and contrast resolution. In addition, patient-related factors also impact the final quality of the image, such as motion artifacts, which appear as unsharpness in the reconstructed image. Moreover, this systematic review aimed to assess the diagnostic accuracy of CBCT scans for VRFs, limited to the identification of the fracture lines. CBCT scans have proven effective in detecting bone defects associated with VRFs, which commonly manifest as J-shaped periradicular lesions, buccopalatal or lingual cortical bone loss, or radiolucent halos in the furcation area [130]. In clinical practice, diagnosing VRFs is often more complex and should involve a thorough assessment that includes radiographic examinations, clinical signs and symptoms, visual inspection, and periodontal evaluation. Additional methods, such as the use of operating microscopes, dyes, or transillumination, can aid in locating fracture lines. A fundamental limitation of in vitro studies is the absence of patient-related factors and clinical manifestations. Therefore, the findings should be interpreted with caution, and further in vivo studies are necessary to validate these results.

Despite the rigorous methodology applied in study selection and risk of bias assessment, the potential influence of publication bias cannot be entirely ruled out. Additionally, the substantial heterogeneity observed among the included studies may limit the generalizability of the findings and should be considered when interpreting the results. The GRADE appraisal revealed a very-low certainty of evidence for the meta-analyses that evaluated the influence of the fracture pattern, voxel size, and intracanal materials. The downgrading occurred because we included only in vitro studies in this systematic review. Many comparisons were performed with indirect evidence and inconsistency, as there was a significant statistical and methodological heterogeneity among the included studies. There was no downgrading due to the risk of bias because rigorous criteria were applied during the quality analysis with QUADAS-2, and studies with high risk were not included in the meta-analyses. Imprecision was also not considered, since there was a significant sample size involved in all included studies. Publication bias or other forms of bias were not detected. Although very strict criteria were used to select studies for the meta-analyses, it is important to consider that this systematic review focused on in vitro studies. Hence, the impact of several aspects regarding fracture patterns and CBCT acquisition were identified.

## 5. Conclusions

The present systematic review identified several factors that influence the diagnostic accuracy of CBCT for detecting VRFs in vitro, including fracture pattern, presence of intracanal materials, and voxel size. The diagnostic accuracy of incomplete VRFs is lower than complete VRFs. Metal posts and root canal fillings also reduce diagnostic accuracy. A minimum voxel size of 0.16 mm is beneficial for the diagnosis of VRFs, in the presence of metal posts. Limited evidence suggests that the use of small FOVs and central positioning of the suspected tooth in the FOV, might improve the diagnostic accuracy. Contrarily, according to the available evidence, current image-enhancement filters and MAR algorithms do not affect VRF diagnosis. However, these findings must be interpreted with caution due to the inherent limitations of in vitro designs, which may not fully replicate clinical conditions. Moreover, the high statistical heterogeneity and the very low certainty of evidence across all meta-analyses further emphasize that future well-designed in vivo studies are essential to validate these results. Other factors, such as variations in tube current and kilovoltage, the presence of metallic objects within the FOV, and the differences in CBCT devices, should be further evaluated to establish an association with the diagnosis of VRFs. Additional strategies and tools for the diagnosis of VRFs should be developed, especially for incomplete fractures and teeth containing root canal filling materials or metal posts.

## Figures and Tables

**Table 1:** Summary table of the sensitivity and specificity values of high and low resolution CBCT images, according to the selected threshold for high resolution, and intracanal materials, and their respective confidence interval.

Voxel size threshold	Resolution	Intracanal material		
		No filling	Root canal filling	Metal post
0.2 mm	High resolution	SSe = 0.768; 95% CI, 0.66–0.85	SSe = 0.715; 95% CI, 0.634–0.785	SSe = 0.572; 95% CI, 0.492–0.65
		SSp = 0.832; 95% CI, 0.772–0.879	SSp = 0.797; 95% CI, 0.736–0.846	SSp = 0.716; 95% CI, 0.648–0.774
	Low resolution	SSe = 0.781; 95% CI, 0.415–0.947	SSe = 0.641; 95% CI, 0.474–0.78	SSe = 0.564; 95% CI, 0.407–0.709
		SSp = 0.883; 95% CI, 0.722—0.956	SSp = 0.919; 95% CI, 0.692–0.983	SSp = 0.615; 95% CI, 0.456–0.753

0.16 mm	High resolution	SSe = 0.708; 95% CI, 0.573–0.814	SSe = 0.716; 95% CI, 0.622–0.794	SSe = 0.632; 95% CI, 0.539–0.716
		SSp = 0.865; 95% CI, 0.764–0.927	SSp = 0.803; 95% CI, 0.724–0.864	SSp = 0.689; 95% CI, 0.611–0.758
	Low resolution	SSe = 0.772; 95% CI, 0.635–0.869	SSe = 0.715; 95% CI, 0.619–0.795	SSe = 0.482; 95% CI, 0.394–0.571
		SSp = 0.868; 95% CI, 0.793–0.918	SSp = 0.833; 95% CI, 0.731–0.902	SSp = 0.749; 95% CI, 0.644–0.832

0.125 mm	High resolution	SSe = 0.72; 95% CI, 0.562–0.837	SSe = 0.742; 95% CI, 0.626–0.831	SSe = 0.665; 95% CI, 0.551–0.762
		SSp = 0.896; 95% CI, 0.834—0.936	SSp = 0.827; 95% CI, 0.768—0.874	SSp = 0.692; 95% CI, 0.593—0.776
	Low resolution	SSe = 0.749; 95% CI, 0.635–0.836	SSe = 0.694; 95% CI, 0.627–0.753	SSe = 0.502; 95% CI, 0.425–0.578
		SSp = 0.813; 95% CI, 0.683–0.897	SSp = 0.792; 95% CI, 0.666–0.88	SSp = 0.735; 95% CI, 0.649–0.806

0.1 mm	High resolution	SSe = 0.763; 95% CI, 0.569–0.888	SSe = 0.686; 95% CI, 0.588–0.771	SSe = 0.654; 95% CI, 0.539–0.753
		SSp = 0.881; 95% CI, 0.832—0.917	SSp = 0.802; 95% CI, 0.746–0.848	SSp = 0.702; 95% CI, 0.605–0.785
	Low resolution	SSe = 0.717; 95% CI, 0.567–0.83	SSe = 0.707; 95% CI, 0.633–0.771	SSe = 0.517; 95% CI, 0.445–0.588
		SSp = 0.855; 95% CI, 0.718–0.932	SSp = 0.795; 95% CI, 0.67–0.881	SSp = 0.734; 95% CI, 0.649–0.805

Abbreviations: CI, confidence interval; SSe, summary sensitivity; SSp, summary specificity.

**Table 2:** GRADE's summary-of-findings table.

Outcomes	Risk of bias	Indirectness	Inconsistency	Imprecision	Publication bias	Other bias	Certainty of the evidence (grade)
Type of fracture							
Complete × incomplete	Not serious	Very serious^a^	Serious^b^	Not serious	Undetected	Undetected	⨁◯◯◯ Very low^a,b^
Voxel size							
High resolution × low resolution	Not serious	Very serious^a^	Serious^b^	Not serious	Undetected	Undetected	⨁◯◯◯ Very low^a,b^
Intracanal materials							
No fillings × root canal filling	Not serious	Very serious^a^	Serious^b^	Not serious	Undetected	Undetected	⨁◯◯◯ Very low^a,b^
No fillings × metal posts	Not serious	Very serious^a^	Serious^b^	Not serious	Undetected	Undetected	⨁◯◯◯ Very low^a,b^
No fillings × fiberglass posts	Not serious	Very serious^a^	Serious^b^	Not serious	Undetected	Undetected	⨁◯◯◯ Very low^a,b^

^a^Because of the in vitro methodology.

^b^High statistical and methodological heterogeneity.

## Data Availability

Additional supporting information can be found online on the https://repositorio.ufsc.br/handle/123456789/74645 (Repositório Institucional UFSC). Other data will be provided upon request.

## Supporting Information

Supporting Information 1 Database search strategy.

Supporting Information 2 Summary of descriptive characteristics of included studies (*n* = 100).

Supporting Information 3 QUADAS-2 questions adapted to this systematic review of in vitro studies.

Supporting Information 4 Excluded articles and reasons for exclusion.
