# Supplementary material for: Exploring Diagnostic Reliability of CBCT for Vertical Root Fractures: A Systematic Review and Meta-Analytical Approach
Source: Int J Dent. 2025 Jul 21;2025:8824867. doi: 10.1155/ijod/8824867 (PMC12303641; doi:10.1155/ijod/8824867)
Supplement: Supporting Information 1 — Database search strategy. [file 8824867.f1.docx]

**SUPPLEMENTARY MATERIAL 1** Database search strategy.

| **Database** | **Search** |
| --- | --- |
| **PUBMED** | ("cone-beam computed tomography"[MeSH Terms] OR "cone beam" OR "cone-beam" OR "cbct" OR "volume computed tomography" OR "volumetric ct" OR "volumetric computed tomography" OR "volume ct" OR "imaging examinations" OR "imaging examination" OR "imaging modalities" OR "imaging modality" OR "diagnostic imaging"[MeSH Terms] OR "diagnostic imaging" OR "imaging modes" OR "imaging mode") AND ("tooth fractures"[MeSH Terms] OR "tooth fractures" OR "tooth fracture" OR "teeth fractures" OR "teeth fracture" OR "root fractures" OR "root fracture" OR "dental fracture" OR "dental fractures" OR "cracked tooth" OR "cracked tooth syndrome"[MeSH Terms] OR "cracked tooth syndrome" OR "cracked teeth" OR "dental cracks") |
| **EMBASE** | ('cone beam' OR 'cone-beam' OR 'cbct' OR 'volume computed tomography'/exp OR 'volume computed tomography' OR 'volumetric ct'/exp OR 'volumetric ct' OR 'volumetric computed tomography'/exp OR 'volumetric computed tomography' OR 'volume ct'/exp OR 'volume ct' OR 'imaging examinations' OR 'imaging examination' OR 'imaging modalities' OR 'imaging modality' OR 'diagnostic imaging'/exp OR 'diagnostic imaging' OR 'imaging modes' OR 'imaging mode') AND ('tooth fractures'/exp OR 'tooth fractures' OR 'tooth fracture'/exp OR 'tooth fracture' OR 'teeth fractures' OR 'teeth fracture' OR 'root fractures' OR 'root fracture'/exp OR 'root fracture' OR 'dental fracture'/exp OR 'dental fracture' OR 'dental fractures' OR 'cracked tooth' OR 'cracked tooth syndrome'/exp OR 'cracked tooth syndrome' OR 'cracked teeth' OR 'dental cracks') |
| **SCOPUS** | (TITLE-ABS-KEY("cone beam" OR "cone-beam" OR "cbct" OR "volume computed tomography" OR "volumetric ct" OR "volumetric computed tomography" OR "volume ct" OR "imaging examinations" OR "imaging examination" OR "imaging modalities" OR "imaging modality" OR "diagnostic imaging" OR "imaging modes" OR "imaging mode" ) AND ALL ( "tooth fractures" OR "tooth fracture" OR "teeth fractures" OR "teeth fracture" OR "root fractures" OR "root fracture" OR "dental fracture" OR "dental fractures" OR "cracked tooth" OR "cracked tooth syndrome" OR "cracked teeth" OR "dental cracks" ) ) |
| **WEB OF SCIENCE** | (“cone beam” OR “cone-beam” OR “cbct” OR “volume computed tomography” OR “volumetric ct” OR “volumetric computed tomography” OR “volume ct” OR “imaging examinations” OR “imaging examination” OR “imaging modalities” OR “imaging modality” OR “diagnostic imaging” OR “imaging modes” OR “imaging mode”) AND (“tooth fractures” OR “tooth fracture” OR “teeth fractures” OR “teeth fracture” OR “root fractures” OR “root fracture” OR “dental fracture” OR “dental fractures” OR “cracked tooth” OR “cracked tooth syndrome” OR “cracked teeth” OR “dental cracks”) |
| **LILACS** | (tw:("cone beam" OR "cone-beam" OR "feixe cônico" OR "feixe-cônico" OR "haz cónico" OR "haz-cónico" OR "cbct" OR "tcfc" OR "tchc" OR "volume computed tomography" OR "volumetric computed tomography" OR "tomografia computadorizada volumétrica" OR "volumetric ct" OR "volume ct" OR "tac volumétrico" OR "tac volumétrica" OR "tc volumétrica" OR "tc volumétrico" OR "imaging examinations" OR "imaging examination" OR "imaging modalities" OR "imaging modality" OR "diagnostic imaging" OR "diagnóstico por imagem" OR "imageamento" OR "imageologia" OR "imagiologia" OR "radiodiagnóstico" OR "Imagen Clínica" OR "imaging modes" OR "imaging mode" OR "modo de imagem" OR "modos de imagem" OR "modos de imagens" OR "modo de imagens" OR "modo de imagen" OR "modos de imagen" OR "modo de imágenes")) AND (tw:("tooth fractures" OR "tooth fracture" OR "teeth fractures" OR "teeth fracture" OR "dental fracture" OR "dental fractures" OR "fratura dental" OR "fratura dentária" OR "fraturas dentárias" OR "fraturas dos dentes" OR "fraturas de dentes" OR "fratura de dente" OR "fracturas de los dientes" OR "fractura dental" OR "fracturas dentales" OR "fractura de diente" OR "root fractures" OR "root fracture" OR "fratura radicular" OR "fraturas radiculares" OR "fratura da raiz" OR "fraturas das raízes" OR "fractura de raíz" OR "fractura de raíces" OR "fractura radicular" OR "fracturas radiculares" OR "cracked tooth" OR "cracked tooth syndrome" OR "cracked teeth" OR "dente quebrado" OR "síndrome de dente quebrado" OR "síndrome de diente fisurado" OR "dente trincado" OR "dente fissurado" OR "dente gretado" OR "diente roto" OR "diente quebrado")) |
| **PROQUEST DISSERTATIONS & THESES** | (“cone beam” OR “cone-beam” OR “cbct” OR “volume computed tomography” OR “volumetric ct” OR “volumetric computed tomography” OR “volume ct” OR “imaging examinations” OR “imaging examination” OR “imaging modalities” OR “imaging modality” OR “diagnostic imaging” OR “imaging modes” OR “imaging mode”) AND (“tooth fractures” OR “tooth fracture” OR “teeth fractures” OR “teeth fracture” OR “root fractures” OR “root fracture” OR “dental fracture” OR “dental fractures” OR “cracked tooth” OR “cracked tooth syndrome” OR “cracked teeth” OR “dental cracks”) |
| **LIVIVO** | (“cone beam” OR “cone-beam” OR “cbct” OR “volume computed tomography” OR “volumetric ct” OR “volumetric computed tomography” OR “volume ct” OR “imaging examinations” OR “imaging examination” OR “imaging modalities” OR “imaging modality” OR “diagnostic imaging” OR “imaging modes” OR “imaging mode”) AND (“tooth fractures” OR “tooth fracture” OR “teeth fractures” OR “teeth fracture” OR “root fractures” OR “root fracture” OR “dental fracture” OR “dental fractures” OR “cracked tooth” OR “cracked tooth syndrome” OR “cracked teeth” OR “dental cracks”) |
| **GOOGLE SCHOLAR** | (“cone beam” OR “cone-beam” OR “cbct” OR “volume computed tomography” OR “volumetric ct” OR “volumetric computed tomography” OR “volume ct” OR “imaging examinations” OR “imaging examination” OR “imaging modalities” OR “imaging modality” OR “diagnostic imaging” OR “imaging modes” OR “imaging mode”) AND (“tooth fractures” OR “tooth fracture” OR “teeth fractures” OR “teeth fracture” OR “root fractures” OR “root fracture” OR “dental fracture” OR “dental fractures” OR “cracked tooth” OR “cracked tooth syndrome” OR “cracked teeth” OR “dental cracks”) |
