# Supplementary material for: Exploring Diagnostic Reliability of CBCT for Vertical Root Fractures: A Systematic Review and Meta-Analytical Approach
Source: Int J Dent. 2025 Jul 21;2025:8824867. doi: 10.1155/ijod/8824867 (PMC12303641; doi:10.1155/ijod/8824867)
Supplement: Supporting Information 2 — Summary of descriptive characteristics of included studies (n = 100). [file 8824867.f2.docx]

SUPPLEMENTARY MATERIAL 2 Summary of descriptive characteristics of included studies (n=95).

| **Author, Year;** | **Sample size/tooth type** | **Method of VRF induction** | **Type of fracture** | **Groups and subgroups** | **CBCT device** | **Acquisition parameters** | **Application of filters/ algorithms** | **Root canal conditions** | **Simulation of in vivo conditions** | **Findings** | | **Main conclusions** |
| --- | --- | --- | --- | --- | --- | --- | --- | --- | --- | --- | --- | --- |
|  |  |  |  |  |  |  |  |  |  | **Sens** | **Spec** |  |
| Abdinian et al 2016  Iran | 120 mandibular teeth  (60 premolars  60 molars) | hammer and pin | Unclear | According to the root canal conditions (n=40):  A) no filling  B) root canal filling (gutta-percha and sealer)  C) root canal filling + metal post + cement | Cranex 3D (SoredexOy, Tuusula, Finland) | 89 kVp,  6 mA  FOV: 8x4 cm  Voxel size: 0.2mm | None | A) no filling  B) root canal filling (gutta-percha and sealer)  C) root canal filling + metal post + cement | Dry human mandible | A1) 1.00  A2) 0.80  A3)0.70 | A1) 0.90  A2) 0.60  A3) 0.65 | No significant difference between CBCT and a set of three DPRs with different angulations for VRF detection in posterior teeth. |
| Al Hadi et al 2020  United Arab Emirates | 60 single-rooted teeth (lower premolars) | hammer and nail; UTM | Complete and incomplete (varied sample) | According to the root canal conditions (n=45):  A) no filling  B) root canal filling (gutta-percha and sealer) | CS 9000 3D (Carestream Dental, Rochester, USA) | 60 kVp  5 mA  FOV: 3.7x5 cm  Voxel size: 0.076mm | None | A) no filling  B) root canal filling (gutta-percha and sealer) | None. | A) 1.00  B) 0.933 | A) 1.00  B) 1.00 | CBCT presented higher sensitivity in detection of VRFs in comparison with periapical radiographs. |
| Almeshari et al 2023  Saudi Arabia | 66 single-rooted teeth | hammer and pin | Complete | According to the kilovoltage (kVp):  A) 70  B) 80  C) 90  According to the imaging mode:  1) standard  2) low MAR algorithm  3) mid MAR algorithm  4) high MAR algorithm | Promax 3D (Planmeca, Helsinki, Finland) | A) 70 kVp  B) 80 kVp  C) 90 kVp  10 mA  FOV: 8x5 cm  Voxel size: 0.15mm | Promax 3D MAR algorithm | NR | bovine rib sockets | A1) 0.64  A2) 0.64  A3) 0.33  A4) 0.32  B1) 0.47  B2) 0.56  B3) 0.50  B4) 0.58  C1) 0.61  C2) 0.64  C3) 0.36  C4) 0.50 | A1) 0.91  A2) 0.89  A3) 0.94  A4) 0.94  B1) 0.94  B2) 0.92  B3) 0.92  B4) 0.86  C1) 0.89  C2) 0.98  C3) 0.82  C4) 0.94 | Using low MAR at 90 kVp significantly increased the accuracy within the group of 90 kVp. In contrast, mid MAR and high MAR in 70 and 90 kVp, respectively, decreased accuracy significantly. |
| Amintavakoli, 2013  Canada | 30 teeth (incisors, premolars, and molars) | bench vise | Incomplete | According to the voxel size:  A) 0.076-mm  B) 0.1 mm  C) 0.2 mm  D) 0.3 mm | Kodak 9000 3D (Kodak Dental Systems, Carestream Health, Rochester, USA) | 65 kVp  2.5 mA  FOV: NR  Voxel size:  A) 0.076mm  B) 0.1 mm  C) 0.2 mm  D) 0.3 mm | None | no filling | Gypsum stone blocks | A) 0.64  B) 0.667  C) 0.613  D) 0.507 | A) 0.706  B) 0.76  C) 0.706  D) 0.746 | The 0.1-mm voxel size may be the most optimal for this task balancing image noise and contrast. |
| Ardakani et al 2015  Iran | 80 single-rooted teeth | Hammer and chisel | Incomplete | None. | Promax 3D (Planmeca, Helsinki, Finland) | 66 kVp  8 mA  FOV: 8x8 cm  Voxel: NR | None. | no filling | Dry human mandible and skull | 0.975 | 0.95 | The accuracy of CBCT in detection of vertical root fracture are  higher than periapical radiography. |
| Bahmani et al. 2021  Iran | 100 mandibular premolars | UTM | Unclear | According to the root filling material:  A) gutta-percha + sealer  B) bioceramic root filling material | Cranex 3D (SoredexOy, Tuusula, Finland) | 90 kVp  10 mA  FOV: 6x8 cm  Voxel size: 0.2mm | None | A) gutta-percha + sealer  B) bioceramic root filling material | Acrylic blocks | A) 0.84  B) 0.93 | A) 0.78  B) 0.95 | The accuracy of CBCT in detection of vertical root fracture was higher with the bioceramic root filling material. |
| Barros-Costa et al., 2025  Brazil | 22 single-rooted teeth | UTM | Unclear | According to the clinical conditions:  A) no filling and no implant adjacent  B) no filling with implant adjacent  C) gutta-percha and no implant adjacent  D) gutta-percha with implant adjacent  According to the method of postacquisition reconstruction:  1) Filtered-Back Projection  2) Iterative Reconstruction  According to the use of metal artifact reduction algorithm:  a) no MAR  b) Midmark EIOS MAR | Midmark EIOS (Midmark, Dayton, USA) | 84 kVp  12 mA  FOV: 5x5cm  Voxel size: 0.078mm | Midmark EIOS metal artifact reduction algorithm | A) no filling  B) gutta-percha | Dry human mandible | a)  A1) 0.68  A2) 0.76  B1) 0.76  B2) 0.72  C1) 0.68  C2) 0.50  D1) 0.64  D2) 0.50  b)  A1) 0.68  A2) 0.80  B1) 0.70  B2) 0.66  C1) 0.44  C2) 0.44  D1) 0.54  D2) 0.42 | a)  A1) 0.73  A2) 0.88  B1) 0.48  B2) 0.63  C1) 0.63  C2) 0.77  D1) 0.58  D2) 0.65  b)  A1) 0.77  A2) 0.88  B1) 0.67  B2) 0.83  C1) 0.75  C2) 0.88  D1) 0.80  D2) 0.83 | The reconstruction method did not significantly influence VRF detection. |
| BashizadehFakhar et al. 2021  Iran | 60 single-rooted teeth | Hammer and pin | Unclear | According to the acquisition parameter (kilovoltage):  A) 80 kVp  B) 92 kVp | Promax 3D (Planmeca, Helsinki, Finland) | A) 80 kVp  B) 92 kVp  4 mA  FOV: 5,5x10 cm  Voxel size: 0.15mm | None | Root canal filling (gutta-percha + sealer) and metal post | Bovine rib sockets | A) 0.86  B) 0.6 | A) 0.333  B) 0.3 | The tube voltage of 80 kVp were more efficient in the diagnosis VRF. |
| Bechara et al. 2013a  United States | 66 single-rooted teeth | Hammer and pin | Complete | According to the CBCT device:  A) Promax Planmeca  B) Picasso Master 3D | A) Promax 3D (Planmeca, Helsinki, Finland)  B) Picasso Master 3D (EWOO technology, Republic of Korea) | kVp: NR  mA: NR  FOV:  A) 8x8 cm  B) 16x7 cm  Voxel size: 0.2mm | None. | Root canal filling (gutta-percha) | Bovine rib sockets | A) 0.81  B) 0.61 | A) 0.78  B) 0.61 | CBCT small FOVs presented the most favorable results. |
| Bechara et al. 2013b  United States | 66 single-rooted teeth | Hammer and pin | Complete | According to the CBCT device:  A) Promax Planmeca  B) Picasso Master 3D  According to the imaging mode:  1) original images  2) MAR algorithm | A) Promax 3D (Planmeca, Helsinki, Finland)  B) Picasso Master 3D (EWOO technology, Republic of Korea) | kVp: NR  mA: NR  FOV:  A) 8x8 cm  B) 16x7 cm  Voxel size: 0.2mm | A) Promax Planmeca MAR algorithm  B) Picasso Master 3D MAR algorithm | Root canal filling (gutta-percha) | Bovine rib sockets | *  A1) 0.80  A2) 0.71  B1) 0.61  B2) 0.51 | *  A1) 0.78  A2) 0.59  B1) 0.61  B2) 0.54 | MAR decreased the accuracy of RF detection in endodontically  treated teeth. |
| Bechara et al. 2013c  United States | 66 single-rooted teeth | Hammer and pin | Complete | According to the scan mode:  A) 180°  B) 360° | Accuitomo 3D (J. Morita, Kyoto, Japan) | 76 kVp  6 mA  FOV: 6x6 cm  Voxel size: 0.125mm | None. | Root canal filling (gutta-percha) | Bovine rib sockets | *  A) 0.7  B) 0.65 | *  A) 0.6  B) 0.74 | Only the specificity is improved by the 360° scan. |
| Bezerra et al. 2015  Brazil | 30 single rooted teeth | UTM | Complete and incomplete | According to the type of fracture:  A) Complete  B) Incomplete  According to the imaging mode:  1) original images  2) ARA | Picasso Trio 3D imaging system (Vatech, Hwaseong, Republic of Korea) | 90 kVp  5 mA  FOV: 8x5 cm  Voxel size: 0.2mm | Picasso Trio MAR algorithm | metal post | Dry human mandible | A1) 0.483  A2) 0.483  B1) 0.450  B2) 0.383 | A1) 0.583  A2) 0.433  B1) 0.583  B2) 0.433 | ARA had a negative impact on the diagnosis |
| Brady et al. 2014  United Kingdom | 30 mandibular teeth  14 premolars  16 molars | UTM | Complete and Incomplete | According to the type of fracture:  A) Complete VRF  B) Incomplete VRF  According to the CBCT device:  1) 3D Accuitomo  2) i-CAT | 1) Accuitomo 3D (J. Morita, Kyoto, Japan)  2) i-CAT Next Generation (Imaging Sciences International, Hatfield, PA) | 1) 90 kV  3 mA  FOV: 4x4 cm  Voxel size: 0.08mm  2) 120 KV  5 mA  FOV: 16x4 cm  Voxel size: 0.125-mm | None | no filling | Dry human mandible | A1) 0.98  A2) 0.98  B1) 0.27  B2) 0.28 | A1) 1.00  A2) 0.99  B1) 0.99  B2) 1.00 | The detection  of complete fractures was significantly higher for all  systems than that of incomplete fractures. |
| Bragatto et al. 2016  Brazil | 20 teeth (premolars) | UTM | Unclear | According to the voxel size:  A) 0.125 mm  B) 0.2 mm  C) 0.25 mm  D) 0.3 mm  E) 0.4 mm | i-CAT (Imaging Sciences International, Hatfield, PA) | 120 kVp  FOV: 8x8 cm  A) 37.07 mA  B) 37.07 mA  C) 37.07 mA  D) 18.54 mA  E) 18.54 mA  Voxel size:  A) 0.125 mm  B) 0.2 mm  C) 0.25 mm  D) 0.3 mm  E) 0.4 mm | None.. | no filling | Dry human mandible | A) 1.0  B) 1.0  C) 1.0  D) 0.97  E) 0.97 | A) 1.0  B) 1.0  C) 0.9  D) 0.5  E) 0.27 | Voxel size 0.125 mm produced images with the best resolution. Voxel sizes of 0.3 and 0.4 should be avoided. |
| Byakova et al. 2019  Russia | 50 single-rooted teeth | hammer and pin | **Complete and Incomplete | According to the fracture width:  A) Incomplete  B) Complete | Accuitomo 3D (J. Morita, Kyoto, Japan) | 90 kVp  4-5 mA  FOV: 8х8mm Voxel size: 0.16mm | None | metal post | Acrylic blocks | A) 0.27  B) 0.53 | A) 0.56  B) 0.62 | Fracture width affected the in vitro detectability.  The detectability in vivo  was decreased because of low image quality. |
| Caetano et al. 2020  Brazil | 45 single-rooted teeth | **UTM | **Complete and incomplete (varied sample) | According to the root canal conditions:  A) no filling  B) root canal filling (gutta-percha + sealer)  C) root canal filling + metal post  According to the CBCT device:  1) Prexion 3D  2) OP300  3) 9000 3D  According to the imaging mode and software:  a) original images, In Vivo Dental  b) AR filter, e-Vol DX | 1) Prexion 3D (Yoshida Dental, Tokyo. Japan)  2) OP300 Maxio (Instrumentarim Dental, Tuusula, Finland)  3) Kodak 9000 3D (Kodak Dental Systems, Carestream Health, Rochester, NY, EUA) | 1) 90 kVp  4 mA  FOV: 5.1x5.1 cm  Voxel size: 0.1mm  2) 90 kVp  10 mA  FOV: 6x4 cm  Voxel size: 0.085mm  3) 70 kVp  10 mA  FOV: 5x3.7 cm  Voxel size: 0.076mm | Blooming Artifact Reduction filter (BAR), e-Vol DX | A) no filling  B) root canal filling (gutta-percha + sealer)  C) root canal filling + metal post | Dry human mandible | *  a)  A1) 1.00  A2) 0.80  A3) 0.67  B1) 0.96  B2) 0.83  B3) 0.40  C1) 0.93  C2) 0.80  C3) 0.73  b)  A1) 1.00  A2) 0.76  A3) 0.63  B1) 1.00  B2) 0.93  B3) 0.63  C1) 0.93  C2) 0.86  C3) 0.63 | *  a)  A1) 0.93  A2) 0.90  A3) 0.63  B1) 1.00  B2) 0.86  B3) 0.70  C1) 0.93  C2) 0.96  C3) 0.60  b)  A1) 0.83  A2) 0.80  A3) 0.90  B1) 0.90  B2) 0.90  B3) 0.70  C1) 0.83  C2) 0.93  C3) 0.93 | The PreXion 3D device is the most accurate when detecting VRF. |
| Candemil et al. 2020  Brazil | 20 single-rooted teeth | UTM | **Complete | According to the CBCT unit:  A) CS 9300  B) ProMax  C) NewTom  According to the presence of metallic objects in the exomass or endomass:  1) None.  2) one object in the exomass  3) two objects in the exomass  4) one object in the exomass and one in the endomass  5) one object in the endomass | A) CS 9300 (Carestream Dental, Rochester, NY)  B) Promax 3D (Planmeca, Helsinki, Finland)  C) NewTom VG (Quantitative Radiology, Verona, Italy) | A) 90 kVp  100 mA  FOV: 5x5  Voxel size: 0.09mm  B) 90 kVp  96 mA  FOV: 4.5x4.5  Voxel size:0.1mm  C) 90 kVp  44.8 mA  FOV: 6x7 cm  Voxel size: 0.08mm | None | fiberglass post | Dry human mandible | *média  A1) 0.9  A2) 0.915*  A3) 0.9*  A4) 0.915*  A5) 0.94*  B1) 0.98  B2) 0.965*  B3) 0.965*  B4) 0.925*  B5) 0.9*  C1) 0.95  C2) 0.975*  C3) 0.95*  C4) 0.915*  C5) 0.88* | *média  A1) 0.93  A2) 0.955*  A3) 0.95*  A4) 0.88*  A5) 0.89*  B1) 0.95  B2) 1.00*  B3) 0.95*  B4) 0.9*  B5) 0.865*  C1) 0.9  C2) 0.89*  C3) 0.98*  C4) 0.89*  C5) 0.925* | Exomass-related metal artefacts did not influence the diagnosis of simulated VRF in CBCT. |
| Candemil et al., 2021  Brazil | 20 single-rooted teeth | UTM | **Complete | According to the presence of metallic objects in the exomass or endomass:  A) one object in the exomass  B) two objects in the exomass  C) one object in the exomass and one in the endomass  D) one object in the endomass  According to the acquisiton parameters:  1) 90 kVp, 100 mA  2) 70 kVp, 24 mA | CS 9000 3D (Carestream Dental, Rochester, NY) | 1) 90 kVp,  100 mA  2) 70 kVp,  24 mA  FOV: 5x5 cm  Voxel size: 0.09mm | None | fiberglass post | Dry human mandible | A1) 0.90**  A2) 0.77**  B1) 0.88**  B2) 0.74**  C1) 0.84**  C2) 0.71**  D1) 0.89**  D2) 0.64** | A1) 0.96**  A2) 0.82**  B1) 0.96**  B2) 0. 85**  C1) 0.90**  C2) 0.83**  D1) 0.91**  D2) 0.90** | The 70 kVp/24 mA presented higher accuracy regardless of the number of metallic objects in the exomass and endomass. |
| Cavalcanti et al., 2020  Brazil | 200 single-rooted teeth | Hammer and chisel | Complete | According to the presence of root canal filling and endodontic sealer:  A) no filling  B) gutta-percha + Pulp Canal Sealer  C) gutta-percha + AH Plus  D) gutta-percha + Sealer 26  E) gutta-percha + BC Sealer | Cranex 3Dx | 90 kVp  10 mA  0.15 mm voxel FOV: 8x6 cm | None. | A) no filling  B) gutta-percha + Pulp Canal Sealer  C) gutta-percha + AH Plus  D) gutta-percha + Sealer 26  E) gutta-percha + BC Sealer | Gypsum stone blocks | A) 0.0.675  B) 0.575  C) 0. 525  D) 0.45  E) 0.30 | A) 0.775  B) 0.775  C) 0.775  D) 0.90  E) 0.85 | Different endodontic sealers might affect VRF diagnosis. |
| da Silveira et al 2013  Brazil | 60 single-rooted teeth | hammer and chisel | Incomplete | According to the root canal conditions:  A) no filling  B) root canal filling (gutta-percha and sealer)  C) root canal filling + metal post + cement  According to the voxel size:  1) 0.2 mm  2) 0.3 mm  3) 0.4 mm | i-CAT (Imaging Sciences International, Hatfield, PA) | 120 kVp  3-8 mA  FOV: 8x8 cm  Voxel size:  1) 0.2 mm  2) 0.3 mm  3) 0.4 mm | None. | A) no filling  B) root canal filling (gutta-percha and sealer)  C) root canal filling + metal post + cement |  | A1) 0.97  A2) 0.87  A3) 0.76  B1) 0.97  B2) 0.67  B3) 0.6  C1) 0.83  C2) 0.63  C3) 0.57 | A1) 1.00  A2) 0.97  A3) 0.8  B1) 0.93  B2) 0.74  B3) 0.7  C1) 0.8  C2) 0.91  C3) 0.59 | 0.2-voxel presented higher accuracy for teeth with filling and/or a post. |
| Dalili Kajan et al 2018  Iran | 60 single-rooted teeth (premolars) | post turned with a wrench | Incomplete | According to the root canal conditions:  A) root canal filling (gutta-percha and sealer)  B) root canal filling + metal post + cement  According to the imaging mode: algorithm:  1) original images  2) MAR algorithm | Pax-i3D (Orangedental, Biberach an der Riss, Germany) | 95 kVp  6 mA  FOV: 9x12 cm  Voxel size: 0.2mm | Pax-i3D MAR algorithm | A) root canal filling (gutta-percha and sealer)  B) root canal filling + metal post + cement | Gypsum stone blocks | A1) 0.4667  A2) 0.8667  B1) 0.4667  B2) 0.6667 | A1) 0.5333  A2) 0.4  B1) 0.6667  B2) 0.6667 | There were no significant differences between the efficacies of imaging modes. |
| De Lima Moreno et al., 2022  Brazil | 20 single-rooted teeth | Hammer and pin | unclear | According to the CBCT device:  A) OP300  B) Ortophos SL 3D  C) PaX.i-3D  According to the acquisition mode:  1) Standard  2) High resolution  According to the root canal conditions:  a) fiberglass post  b) metal post | A) OP300  B) Ortophos SL 3D  C) PaX.i-3D | A1) 89 kVp  8 mA  0.13-mm voxel  FOV: 4.7x4.7 cm  A2) 89 kVp  10 mA  0.08-mm voxel  FOV: 4.7x4.7 cm  B1) 85 kVp  10 mA  0.16-mm voxel  FOV: 5x5.5 cm  B2) 85 kVp  6 mA  0.08-mm voxel  FOV: 5x5.5 cm  C1) 89 kVp  5 mA  0.13-mm voxel  FOV: 6x6 cm  C2) 89 kVp  5 mA  0.08-mm voxel  FOV: 6x6 cm | None. | a) fiberglass post  b) metal post | Container filled with water | NR | NR | The CBCT device and the root canal conditions affect the diagnosis of VRFs. |
| De Martin e Silva et al. 2018  Brazil | 40 single-rooted teeh | Hammer and chisel | **Complete | According to the root canal conditions:  A) root canal filling (gutta-percha + sealer)  B) root canal filling + metal post  According to the voxel sizes:  a) 0.25 mm  b) 0.3 mm  According to the optimization filters:  1) original images  2) Sharpen filter  3) Hard filter | i-CAT (Imaging Sciences International, Hatfield, PA) | 120 kVp  5 mA  FOV: 6x23 cm  Voxel size: NR | Sharpen; Hard; i-CAT Vision software | A) root canal filling (gutta-percha + sealer)  B) root canal filling + metal post | Dry human mandible | NR | NR | The presence of a metal post and the voxel size significantly interfere with the diagnosis of VRF. Despite the formation of metal artifacts associated with metallic cores, applying filters did not improve the diagnosis. |
| De Menezes et al 2016  Brazil | 48 single-rooted teeth | Hammer and pin | **Incomplete | According to the root canal conditions:  A) no filling  B) gutta-percha  C) metal post | Prexion (Yoshida Dental, Tokyo. Japan) | 90 kV  4 mA  FOV: 5x5 cm  Voxel size:0.1mm | None. | A) no filling  B) gutta-percha  C) metal post | Dry human mandible | *  A) 0.9166  B) 0.6666  C) 0.7083 | *  A) 0.8333  B) 0.8333  C) 0.3333 | The presence of posts and gutta-percha reduced the sensitivity and the accuracy in detecting the VRF. |
| De Rezende Barbosa et al., 2016  Brazil | 44 single-rooted teeth | Hammer and pin | Complete | According to the imaging mode:  A) original images  B) MAR algorithm  According to the root canal condition:  1) no filling  2) gutta-percha  3) metal post  4) fiberglass post | Picasso Trio (Vatech, Hwaseong, Republic of Korea) | 80 kVp  4 mA  **FOV: 12x7 cm  Voxel size: 0.2mm | MAR algorithm (EasyDent4, E-WOO,  Giheung-gu, Republic of Korea) | 1) no filling  2) gutta-percha  3) metal post  4) fiber post | Dry human mandible | A1) 0.89  A2) 0.69  A3) 0.54  A4) 0.83  B1) 0.83  B2) 0.60  B3) 0.52  B4) 0.92 | A1) 0.87  A2) 0.86  A3) 0.75  A4) 0.84  B1) 0.84  B2) 0.84  B3) 0.84  B4) 0.83 | ARA did not influence the diagnosis of root fractures; gold posts reduced the overall CBCT diagnostic ability |
| Dias-Junior et al 2023  Brazil | 20 single-rooted teeth | Complete and incomplete | UTM | According to the type of fracture:  a) complete  b) incomplete  According to the intracanal conditions:  A) no filling  B) gutta-percha  C) fiberglass post  D) metal post  According to the imaging filter:  1) no filter  2) BAR 2 filter | Prexion 3D Elite (Prexion, San Mateo, USA) | 90 kVp  4 mA  FOV: 5x5 cm  Voxel size: 0.09mm | eVOL DXS BAR 2 filter (CDT software, Bauru, Brazil) | A) no filling  B) gutta-percha  C) fiberglass post  D) metal post | Dry human skull | a)  A1) 0.95  A2) 1.0  B1) 0.95  B2) 1.0  C1) 1.0  C2) 1.0  D1) 0.90  D2) 0.90  b)  A1) 0.40  A2) 0.40  B1) 0.40  B2) 0.35  C1) 0.40  C2) 0.25  D1) 0.15  D2) 0.25 | a)  A1) 0.85  A2) 0.90  B1) 0.90  B2) 1.0  C1) 0.95  C2) 1.0  D1) 0.85  D2) 0.90  b)  A1) 0.85  A2) 0.90  B1) 0.90  B2) 1.0  C1) 0.95  C2) 1.0  D1) 0.85  D2) 0.90 | The BAR filter did not improve the diagnostic accuracy of VRFs. The intracanal materials also did not influence the diagnosis. Incomplete VRFs were highly associated with a decrease in sensitivity. |
| Ferreira et al. 2013  Brazil | 60 bi-rooted teeth (maxillary premolars) | Hammer and pin | Incomplete | According to the root canal conditions:  A) root canal filling (gutta-percha and sealer) + fiber post  B) root canal filling + metal post  According to the CBCT unit:  1) i-CAT  2) Scanora 3D | 1) i-CAT (Imaging Sciences International, Hatfield, PA)  2) Scanora 3D (Soredex, Tuusula, Finland) | 1) 120 kVp  36.12 mA  FOV: 6x8 cm  Voxel size: 0.125mm  2) 85 kVp  8 mA  FOV: 6x6 cm  Voxel size: 0.133mm | None. | A) root canal filling (gutta-percha and sealer) + fiber post  B) root canal filling + metal post | Acrylic blocks | A1) 0.85  A2) 0.73  B1) 0.72  B2) 0.73 | A1) 0.74  A2) 0.71  B1) 0.75  B2) 0.76 | The diagnostic performance for detecting vertical fractures was higher for roots with fiber-resin than with  titanium posts. |
| Ferreira et al. 2015  Brazil | 40 single-rooted teeth | UTM | Unclear | According to the imaging mode (application of filters):  A) original images  B) sharpen filter  C) Sharpen mild filter  D) sharpen 3x3 filter  E) S9 filter  F) smooth filter  G) smooth 3x3 filter  According to the root canal conditions:  1) no filling  2) metal post | i-CAT (Imaging Sciences International, Hatfield, PA) | kV:NR  mA: NR  FOV: 8x8 cm  Voxel size:0.2mm | A) original images  B) sharpen filter  C) Sharpen mild filter  D) sharpen 3x3 filter  E) S9 filter  F) smooth filter  G) smooth 3x3 filter | 1) no filling  2) metal post | Dry human mandible | A1) 0.5  A2) 0.2  B1) 0.48  B2) 0.32  C1) 0.75  C2) 0.28  D1) 0.60  D2) 0.45  E1) 0.55  E2) 0.13  F1) 0.48  F2) 0.18  G1) 0.64  G2) 0.20 | A1) 0.85  A2) 0.87  B1) 0.58  B2) 0.62  C1) 0.63  C2) 0.90  D1) 0.70  D2) 0.65  E1) 0.78  E2) 0.98  F1) 0.88  F2) 0.80  G1) 0.70  G2) 0.93 | The use of enhancement filters in CBCT images has no influence on the  diagnosis of VRFs in teeth with metal posts |
| Fisekcioglu et al. 2014  Turkey | 104 teeth (mixed) | UTM | Complete | None. | ILUMA Ultra (IMTEC Imaging, USA) | 120 kVp  3.8 mA  FOV: 21.1x14.2 cm  Voxel size:0.3mm | None. | no filling | Dry human mandible | 0.838* | 0.996* | Detailed information about root fractures may be obtained using CBCT. |
| Fontenele et al. 2020  Brazil | 30 single-rooted teeth (mandibular premolars) | UTM | **Incomplete | According to the  tube current:  A) 4 mA  B) 8 mA  C) 10 mA  According to the imaging mode:  1) original images  2) MAR algorithm  According to the presence of an adjacent implant:  a) absent  b) present | OP300 Maxio (Instrumentarium Dental, Tuusula, Finland) | 90kVp  mA:  A) 4 mA  B) 8 mA  C) 10 mA  FOV: 5x5 cm  Voxel size: 0.08mm | OP300 MAR algorithm  a) original images  b) MAR algorithm | no filling | Dry human mandible | a)  A1) 0.613  A2) 0.560  B1) 0.500  B2) 0.680  C1) 0.560  C2) 0.560  b)  A1) 0.600  A2) 0.613  B1) 0.627  B2) 0.600  C1) 0.550  C2) 0.654 | a)  A1) 0.653  A2) 0.653  B1) 0.686  B2) 0.746  C1) 0.773  C2) 0.800  b)  A1) 0.573  A2) 0.591  B1) 0.706  B2) 0.627  C1) 0.586  C2) 0.520 | The zirconium implants impair the diagnosis of VRF in adjacent teeth. Neither the tube current nor the MAR tool is effective in improving the diagnosis of VRF. |
| Fox et al. 2018  Canada | 176 mandibular premolars | UTM | **Complete and incomplete (varied sample) | According to the root canal conditions:  A) gutta-percha + sealer  B) zirconium-based cone + sealer | CS 9000 3D (Carestream Dental, Rochester, NY) | 70 kVp  3.2 mA  FOV:5x3.7 cm  Voxel size: 0.076mm | None. | A) gutta-percha + sealer  B) zirconium-based cone + sealer | Dry human mandible | A) 0.4659  B) 0.5833 | A) 0.8485  B) 0.7386 | The Zr group improved the sensitivity of the detection  of artificially induced VRFs. |
| Freitas et al 2019  Brazil | 20 single-rooted teeth | UTM | Incomplete | Implant adjacent and MAR algorithm:  A) without implant and MAR  B) with implant and without MAR  C) with implant and MAR  According to the kilovoltage:  1) 70 kVp  2) 80 kVp  3) 90 kVp | Promax 3D (Planmeca, Helsinki, Finland) | 1) 70 kVp  2) 80 kVp  3) 90 kVp  10 mA  FOV: 8x5 cm  Voxel size: 0.15mm | ProMax 3D MAR tool | no filling | Dry human mandible | A1) 0.564  A2) 0.558  A3) 0.495  B1) 0.614  B2) 0.577  B3) 0.558  C1) 0.577  C2) 0.545  C3) 0.501 | A1) 0.737  A2) 0.815  A3) 0.896  B1) 0.658  B2) 0.707  B3) 0.777  C1) 0.676  C2) 0.832  C3) 0.839 | Artifacts produced in the vicinity of teeth with suspected VRF impaired the diagnosis by decreasing the specificity, which was improved using MAR. |
| Freitas-e-Silva et al. 2019  Brazil | 80 single-rooted premolars | UTM | **Complete and incomplete (varied sample) | According to the CBCT unit:  A) i-CAT  B) Orthophos XG  C) Prexion 3D | A) i-CAT (Imaging Sciences International, Hatfield, PA)  B) Orthophos XG (Sirona, Bensheim, Germany)  C)Prexion 3D (Yoshida Dental, Tokyo. Japan) | A) 120 kV  5 mA  FOV: 6x6 cm  Voxel size: 0.2mm  B) 85 kV  6 mA  FOV: 5x5 cm  Voxel size: 0.16mm  C) 90 kV  4 mA  FOV: 5x5 cm  Voxel size: 0.075mm | None. | gutta-percha | Acrylic blocks | A) 0.93  B) 0.73  C) 0.88 | 1) 0.70  2) 0.73  3) 0.75 | Endodontic sealers did not influence the detection of VRF. The PreXion device was the most accurate,  having the highest specificity value. |
| Gaêta-Araujo et al. 2017  Brazil | 20 single-rooted teeth | UTM | **Complete | According to:  Intracanal material:  A) no filling  B) gutta-percha  C) metal post  D) fiberglass post  According to the tube current:  1) 4 mA  2) 8 mA  3) 10 mA  4) 13 mA | OP300 Maxio (Instrumentarium Dental, Tuusula, Finland) | 90 kVp  1) 4 mA  2) 8 mA  3) 10 mA  4) 13 mA  FOV: 4x6 cm  Voxel size: 0.085mm | None. | A) no filling  B) gutta-percha  C) metal post  D) fiberglass post | Dry human mandible | A1) 0.59  A2) 0.69  A3) 0.62  A4) 0.64  B1) 0.54  B2) 0.60  B3) 0.66  B4) 0.54  C1) 0.47  C2) 0.46  C3) 0.50  C4) 0.43  D1) 0.68  D2) 0.70  D3) 0.56  D4) 0.60 | A1) 0.72  A2) 0.80  A3) 0.84  A4) 0.82  B1) 0.60  B2) 0.67  B3) 0.60  B4) 0.62  C1) 0.50  C2) 0.72  C3) 0.80  C4) 0.66  D1) 0.68  D2) 0.70  D3) 0.82  D4) 0.80 | For teeth with gutta-percha and metal post, an increased milliampere may lead to increased diagnostic performance of VRF. |
| Gaêta-Araujo et al. 2020  Brazil | 10 single-rooted teeth (mandibular premolars) | UTM | **Incomplete | According to the presence of metal posts in adjacent teeth:  a) None.  b) one adjacent tooth  c) both adjacent teeth  According to the tube current:  A) 4 mA  B) 8 mA  C) 10 mA  According to the imaging mode:  1) original images  2) MAR algorithm | OP300 Maxio (Instrumentarium Dental, Tuusula, Finland) | 90 kVp  FOV: 5x5 cm  Voxel size: 0.125mm  A) 4 mA  B) 8 mA  C) 10 mA | OP300 MAR algorithm | No filling | Dry human mandible | a)  A1) 0.76  A2) 0.70  B1) 0.66  B2) 0.75  C1) 0.56  C2) 0.72  b)  A1) 0.62  A2) 0.66  B1) 0.64  B2) 0.64  C1) 0.66  C2) 0.64  c)  A1) 0.44  A2) 0.64  B1) 0.62  B2) 0.44  C1) 0.54  C2) 0.54 | a)  A1) 0.40  A2) 0.32  B1) 0.38  B2) 0.48  C1) 0.50  C2) 0.42  b)  A1) 0.32  A2) 0.36  B1) 0.38  B2) 0.52  C1) 0.52  C2) 0.54  c)  A1) 0.34  A2) 0.48  B1) 0.64  B2) 0.52  C1) 0.54  C2) 0.58 | The presence of both adjacent teeth restored with a metal post impairs VRF detection; however, an increase in tube current up to 8 mA may aid in this diagnostic task. Moreover, the MAR tool does not seem to be efficient in those cases. |
| Groenke et al 2023  USA­­­­ | 120 teeth | UTM | Complete and incomplete (mixed) | None | CS-9000 (Carestream Dental, Rochester, USA) | 70 kVp  8 mA  FOV: NR  Voxel size: 0.08mm | None. | root canal filling (gutta-percha + endodontic sealer) | None. | 0.58 | 0.87 | No significant difference in sensitivity or specificity between MRI and CBCT in detecting VRF. |
| Gunduz et al. 2013  Turkey | 90 single-rooted teeth | Hammer and pin | Unclear | According to the CBCT device:  A) NewTom 3G  B) 3D Accuitomo 170 | A) NewTom 3G (Quantitative Radiology, Verona, Italy)  B) Accuitomo 3D (J. Morita, Kyoto, Japan) | A) 65 kVp  2 mA  FOV: 4x4 cm  Voxel size: 0.125mm  B) 110 kVp  Automated adjusted mA  FOV: 15x15 cm  Voxel size: 0.16mm | None. | no filling | Dry human mandible | NR | NR | The 3D Accuitomo 170 was significantly superior to the NewTom 3G images in the detection of VRFs. |
| Hassan et al. 2009  Netherlands | 80 teeth  (40 premolars  40 molars) | Hammer and chisel | **Complete | According to the evaluators:  A) Endodontists  B) Dental students  C) Overall | i-CAT (Imaging Sciences International, Hatfield, PA) | 120 KvP  5 mA  FOV: 10x16 cm  Voxel size: 0.25mm | None. | No filling and gutta-percha filled canals (mixed sample) | Dry human mandible | A) 0.775  B) 0.813  C) 0.794  1) 0.80  2) 0.788 | A) 0.913  B) 0.938  C) 0.925  1) 0.975  2) 0.875 | The results showed an overall higher accuracy for CBCT scans than PRs for detecting VRF. |
| Hassan et al. 2010  Netherlands | 80 teeth  (40 premolars  40 molars) | Hammer and chisel | **Complete | According to the CBCT device:  A) NewTom 3G  B) i-CAT  C) Galileos 3D  D) Scanora 3D  E) AccuiTomo-XYZ | A) NewTom 3G (Quantitative Radiology, Verona, Italy)  B) i-CAT (Imaging Sciences International, Hatfield, PA)  C) Galileos 3D (Sirona  Bensheim, Germany)  D) Scanora 3D (Soredex, Tuusula, Finland)  E) AccuiTomo-XYZ (J. Morita, Kyoto, Japan) | A) 110 kVp  2.4 mA  FOV: 10x10 cm  Voxel size: 0.2mm  B) 120 kVp  5 mA  FOV: 10x16 cm  Voxel size: 0.25mm  C) 85 kVp  7 mA  FOV: 15x15 cm  Voxel size: 0.3mm  D) 85 kVp  10 mA  FOV: 7.5x10 cm  Voxel size: 0.2mm  E) 80 kVp  3.3 mA  FOV: 3x4 cm  Voxel size: 0.25mm | None. | No filling and gutta-percha filled canals (mixed sample) | Dry human mandible. | A) 0.304  B) 0.775  C) 0.188  D) 0.575  E) 0.481 | A) 0.95  B) 0.913  C) 0.85  D) 0.85  E) 0.907 | Root canal filling presence reduced specificity in all systems. |
| Hekmatian et al. 2018  Iran | 50 teeth (mandibular premolars) | UTM | Unclear | According to the root canal filling:  A) no filling  B) gutta-percha | Galileos 3D (Sirona  Bensheim, Germany) | 85 kVp  13 mA,  Voxel size: NR  FOV: 5x5.5 cm | None. | A) no filling  B) gutta-percha | Dry human mandible | A) 0.72  B) 0.36* | A) 0.96  B) 0.68 | The intracanal filling materials such as gutta-percha reduce  the diagnostic ability of the vertical root fractures. |
| Hesarkhani et al. 2017  Iran | 30 single-rooted teeth | UTM | Unclear | According to the root canal conditions:  A) root canal filling  B) metal post | NR | NR | None. | A) root canal filling  B) metal post | NR | A) 0.533  B) 0.215* | A) 0.533  B) 0.217* | The presence of intra-canal posts from any of the alloys used in the study significantly reduces the rate and diagnostic sensitivity of the CBCT. |
| Hilgert et al., 2024  Brazil | 30 single-rooted teeth | Hammer and chisel | Incomplete | According to the root canal conditions:  A) no filling  B) root canal filling (gutta-percha and sealer)  C) root canal filling + metal post + cement  According to the CBCT device:  1) Veraviewspocs 3D R100  2) Veraview 3D X800  According to the imaging filter:  a) no filter  b) BAR filter | A) Veraviewspocs 3D R100 (J Morita, Kyoto, Japan)  B) Veraview 3D X800 (J Morita, Kyoto, Japan) | A) 90 kVp  8 mA  FOV: 4x4cm  Voxel size: 0.125mm  B) 95 kVp  8 mA  FOV: 4x4cm  Voxel size: 0.08mm | Blooming artifact reduction (BAR) filter (e-vol DX, CDT software, Bauru, Brazil) | A) no filling  B) root canal filling (gutta-percha and sealer)  C) root canal filling + metal post + cement | Acrylic block | NR | NR | Veraview X800 presented higher diagnosrtic accuracy than the Veraviewspocs 3D R100. The BAR filters had no relevance for the diagnosis of VRFs. |
| Jafarzadeh et al., 2022  Iran | 61 premolars | Hammer and pin | Unclear | According to the image gamma values:  A) 0  B) +10  C) -10 | NewTom VGi | 110 kVp  0.3–65.29 mA  Voxel size: NR  FOV: NR | None | Metal post | Sheep mandible | A) 0.86  B) 0.86  C) 0.86 | A) 1.00  B) 1.00  C) 1.00 | Gamma values did not affect VRF diagnosis. |
| Jin-Hua et al 2023  China | 24 single-rooted teeth | Hammer and pin | Complete | According to the root canal conditions:  A) no filling  B) gutta-percha  C) fiber post  D) titanium post  E) gold-palladium post | 3D Accuitomo 170 (J Morita, Kyoto, Japan) | 90 kVp  5 mA  FOV: 6x6 cm  Voxel size: 0.125mm | None. | A) no filling  B) gutta-percha  C) fiber post  D) titanium post  E) gold-palladium post | Pig mandible | A) 0.917  B) 0.833  C) 0.917  D) 0.75  E) 0.333 | A) 1.0  B) 0.917  C) 0.917  D) 0.833  E) 0.583 | The lowest diagnostic accuracy of VRF was found in the gold-palladium post group, and the difference was statistically significant compared with all other groups. |
| Junqueira et al. 2013  Brazil | 18 single-rooted teeth | Hammer and chisel | Complete | According to the root canal conditions:  A) apical filling (gutta-percha + sealer)  B) apical filling + metal post  According to the voxel sizes:  1) 0.25 mm  2) 0.125 mm | i-CAT (Imaging Sciences International, Hatfield, PA) | 120 kVp  8 mA  FOV: 5x5 cm  Voxel sizes:  1) 0.25 mm  2) 0.125 mm | None. | A) apical filling (gutta-percha + sealer)  B) apical filling + metal post | Dry human mandible | A1) 0.78  A2) 1  B1) 0.67  B2) 0.89 | A1) 0.89  A2) 0.89  B1) 0.56  B2) 0.45 | Voxel size did not  significantly influence the diagnosis of vertical root fractures. |
| Kambungton et al. 2012  Thailand | 60 single-rooted teeth | UTM | Unclear | None. | Veraviewepocs 3D (J. Morita, Kyoto, Japan) | 70 kVp  3 mA  Voxel size: NR  FOV: NR | None. | no filling | Dry human mandible | NR | NR | There was no significant difference between intraoral film, a high-resolution complementary metal oxide semiconductor digital imaging system and CBCT in detecting VRFs. |
| Kamburoglu et al. 2010  Turkey | 60 teeth (mandibular premolars) | Hammer and pin | **Incomplete | According to the CBCT device, and acquisition parameter (voxel size):  A) NewTom 3G  B) Iluma Ultra Cone-Beam, 0.3-mm voxel  C) Iluma Ultra Cone-Beam, 0.1-mm voxel | A) NewTom 3G (Quantitative Radiology, Verona, Italy)  B) ILUMA Ultra (IMTEC Imaging, USA) | A) 110 kVp  FOV: 15x15 cm  Voxel size: 0.19mm  B) 120 kVp  3.8 mA  FOV: 21x14 cm.  Voxel size: 0.3mm  C) 120 kVp  3.8 mA  FOV: 21x14 cm  Voxel size: 0.1mm | None. | Root canal filling (gutta-percha and sealer) at the apical root third. | Dry human mandible | NR | NR | Both ultra-resolution Iluma and NewTom 3G images performed better than low-resolution Iluma |
| Khedmat et al. 2012  Iran | 100 single-rooted teeth | Hammer and pin | Unclear | According to the root canal conditions:  A) no filling  B) gutta-percha | Promax 3D (Planmeca, Helsinki, Finland) | 70 kVp  4 mA  FOV: 8x8 cm  Voxel size: 0.16mm | None. | A) no filling  B) gutta-percha | None. | A) 0.92  B) 0.8 | A) 0.88  B) 0.64 | The presence of gutta-percha reduced the accuracy, sensitivity and specificity of CBCT. |
| Lagos de Melo et al 2023  Brazil | 20 single-rooted teeth | UTM | Incomplete | According to the kilovoltage (kVp):  A) 70  B) 80  C) 90  D) 99  According to the imaging mode:  1) standard  2) MAR algorithm | Picasso Trio (Vatech, E-WOO Technology Co, Yongin, Korea) | A) 70 kVp  B) 80 kVp  C) 90 kVp  D) 99 kVp  3.8 mA  FOV: 5x5 cm  Voxel size: 0.2mm | Picasso Trio MAR algorithm | Root canal filling + metal post | Dry human mandible | A1) 0.34  A2) 0.42  B1) 0.28  B2) 0.375  C1) 0.32  C2) 0.34  D1) 0.38  D2) 0.42 | A1) 0.78  A2) 0.88  B1) 0.88  B2) 0.70  C1) 0.86  C2) 0.74  D1) 0.92  D2) 0.74 | The kilovoltage and MAR algorithm did not affect the diagnostic accuracy of CBCT for VRFs. |
| Liang et al 2022  China | 56 teeth | Temperature cycling (100ºC for 1 mi, -196ºC for 1 min) | Incomplete | According to the CBCT device:  A)  B)  C)  D) | A) NewTom VGi (Quantitative Radiology, Verona, Italy)  B) Promax 3D Max (Planmeca, Helsinki, Finland)  C) Scanora-3D (Soredex, Tuusula, Finland)  D) Kavo 3D Exam (Kavo Dental GmbH, Biberach, Germany) | A) 110 kVp  7.48 mA  FOV: 5x5 cm  Voxel size: 0.1mm  B) 80 kVp  6.3 mA  FOV: 5x5 cm  Voxel size: 0.1mm  C) 90 kVp  10 mA  FOV: 5x5 cm  Voxel size: 0.1mm  D) 120 kVp  5 mA  FOV: 8x8 cm  Voxel size: 0.125mm | None. | No fillings | Acrylic blocks | A) 0.36  B) 0.54  C) 0.34  D) 0.22 | A) 0.83  B) 0.50  C) 1.0  D) 1.0 | The diagnostic accuracy for incomplete VRFs was very low for all the four different CBCT units. |
| Makeeva et al. 2016a  Russia | 25 single-rooted teeth | Post screwed into the root canal | **Complete and incomplete | According to the type of fracture:  A) Complete  B) Incomplete | Accuitomo 3D (J. Morita, Kyoto, Japan) | 80 kVp  4 mA  FOV: 4x4 cm  Voxel size: 0.16mm | None. | no filling | None. | A) 0.96  B) 0.32 | A) 0.96  B) 0.96 | The sensitivity for VRFs with a width > 150 µm is reliably higher than for visualizing VRFs < 150 µm. The specificity is not significantly different. |
| Makeeva et al. 2016b  Russia | **45 single rooted-teeth | Hammer and pin | **Complete and incomplete | According to the type of fracture:  A) Complete  B) Incomplete | Accuitomo 3D (J. Morita, Kyoto, Japan) | 80 kVp  4 mA  FOV: 8x8 cm  Voxel size: 0.16mm | None. | root canal filling | None. | A) 1  B) 0.54 | A) 0.92  B) 0.93 | The detectability of VRFs by CBCT was dependent upon fracture width. |
| Mautone et al., 2025  Brazil | 24 single-rooted teeth | UTM | Unclear | According to the root canal conditions:  A) no filling  B) gutta-percha  C) fiberglass post  D) metal post  According to the acquisition protocol:  1) Protocol 1  2) Protocol 2  3) Protocol 3  4) Protocol 4  5) Protocol 5  6) Protocol 6  7) Protocol 7 | - OP300 Maxio (Instrumentarim Dental, Tuusula, Finland) - Veraview 3D X800 (J Morita, Kyoto, Japan) | 1) OP300 Maxio  90 kVp  3.2 mA  FOV: 5x5cm  Voxel size: 0.125mm  2) OP300 Maxio  90 kVp  8 mA  FOV: 5x5cm  Voxel size: 0.2mm  3) OP300 Maxio  90 kVp  6.3 mA  FOV: 5x5cm  Voxel size: 0.28mm  4) OP300 Maxio  90 kVp  6.3 mA  FOV: 5x5cm  Voxel size: 0.085mm  5) Veraview 3D X800  100 kVp  8 mA  FOV: 4x4cm  Voxel size: 0.08mm  6) Veraview 3D X800  100 kVp  8 mA  FOV: 4x8cm  Voxel size: 0.125mm  7) Veraview 3D X800  100 kVp  8 mA  FOV: 8x4cm  Voxel size: 0.08mm | None. | A) no filling  B) gutta-percha  C) fiberglass post  D) metal post | Dry human mandible | NR | NR | Veraview 3D X800 demonstrated greater  accuracy in the detection of root fractures in roots containing metal posts, while the OP300 showed greater accuracy with the use  of fiberglass posts. |
| Mehralizadeh et al. 2018  Iran | 80 single-rooted teeth (premolars) | Hammer and pin | Unclear | According to the acquisition parameters (mA and kVp)  A) 60 kVp, 6 mA  B) 86 kVp, 6 mA  C) 60 kVp, 10 mA  D) 86 kVp, 10 mA | Rotograph Evo 3D (Villa Sistemi Medicali. Buccinasco, Italy | A) 60 kVp,  6 mA  B) 86 kVp,  6 mA  C) 60 kVp,  10 mA  D) 86 kVp,  10 mA  Voxel size: NR  FOV: NR | None. | Root canal filling (gutta-percha and sealer) | Dry human mandible | NR | NR | kVp min/mA max and kVp min/mA min  settings are suitable for the diagnosis of VRFs |
| Melo et al. 2010  Brazil | 180 single-rooted teeth | Hammer and pin | **Complete | According to the root canal condition:  A) no filling  B) gutta-percha  C) metal post  According to the acquisition parameter (voxel size):  1) 0.3 mm  2) 0.2 mm | i-CAT (Imaging Sciences International, Hatfield, PA) | 120 kVp  3–8 mA  FOV: 8x8 cm  Voxel sizes:  1) 0.3 mm  2) 0.2 mm | None. | A) no filling  B) gutta-percha  C) metal post | Dry human skull | A1) 0.53  A2) 0.83  B1) 0.47  B2) 0.93  C1) 0.53  C2) 0.70 | A1) 0.80  A2) 0.87  B1) 0.70  B2) 0.73  C1) 0.63  C2) 0.66 | The CBCT diagnostic ability was not influenced by the presence of posts or gutta-percha, and the  0.3-mm voxel resolution was not reliable for the investigation of VRFs. |
| Melo et al. 2013  Brazil | 180 single-rooted teeth | Hammer and pin | **Complete | According to the root canal condition:  A) no filling  B) gutta-percha  C) metal post  According to the DICOM viewer software:  1) Dolphin v. 11.5  2) InVivoDental v. 5.0  3) KDIS3D v. 2.1.11  4) Xoran | i-CAT (Imaging Sciences International, Hatfield, PA) | 120 kVp  8 mA  FOV: 8x8 cm  Voxel size: 0.2mm | None. | A) no filling  B) gutta-percha  C) metal post | Dry human skull | A1) 0.71  A2) 0.65  A3) 0.71  A4) 0.73  B1) 0.68  B2) 0.61  B3) 0.63  B4) 0.61  C1) 0.43  C2) 0.41  C3) 0.44  C4) 0.41 | A1) 0.88  A2) 0.87  A3) 0.85  A4) 0.79  B1) 0.81  B2) 0.91  B3) 0.78  B4) 0.91  C1) 0.87  C2) 0.91  C3) 0.91  C4) 0.92 | The diagnosis of VRF does not depend on the software used to reconstruct the image from CBCT. The diagnostic accuracy is significantly reduced for all software when root canals are restored with metallic posts. |
| Menezes et al. 2013  Brazil | 48 single-rooted teeth | UTM | **Incomplete | According to the root canal conditions:  A) no filling  B) root canal filling (gutta-percha + sealer)  C) metal post | Prexion 3D (Yoshida Dental, Tokyo. Japan) | 90kV  4mA  FOV: 5x5 cm  Voxel size: 0.1mm | None. | A) no filling  B) root canal filling (gutta-percha + sealer)  C) metal post | Dry human mandible. | A) 0.875  B) 0.625  C) 0.75 | A) 0.75  B) 0.875  C) 0.375 | CBCT is an excellent tool for the VRF diagnosis. The metal post presence resulted in a high percentage of false positive. |
| Mohammadpour et al. 2014  Iran | 80 single-rooted teeth | UTM | Unclear | According to the root canal conditions:  A) no filling  B) metal post  According to the evaluators:  1) radiologists  2) endodontists  3) overall | NewTom VG (Quantitative Radiology, Verona, Italy) | 110 kVp  13.8 mA  FOV: 8x12 cm  Voxel size: 0.15mm | None. | A) no filling  B) metal post | Acrylic blocks | A1) 0.9392  A2) 0.8704  A3) 0.9418  B1) 0.8125*  B2) 0.925*  B3) 0.8625* | A1) 0.8779  A2) 0.6886  A3) 0.8707  B1) 0.725*  B2) 0.725*  B3) 0.725* | Intracanal posts significantly decreased the VRF diagnostic values of CBCT. |
| Moudi et al. 2014  Iran | 96 teeth (mandibular premolars and molars) | Hammer and pin | Unclear | According to the root canal conditions:  A) no filling  B) gutta-percha  C) gutta-percha + metal post | NewTom 5G (Quantitative Radiology, Verona, Italy) | 110 kVp  mA: NR  FOV: NR  Voxel size: 0.3mm | None. | A) no filling  B) gutta-percha  C) gutta-percha + metal post | None. | A) 0.88  B) 0.94  C) 0.81 | A) 1.0  B) 1.0  C) 1.0 | The CBCT scans revealed a high accuracy in the diagnosis of vertical root fractures; the accuracy did  not decrease in the presence of gutta-percha. |
| Moudi et al. 2015  Iran | 40 teeth (mandibular premolars and molars) | Hammer and pin | Unclear | According to the root canal conditions:  A) gutta-percha  B) metal post  According to the FOV:  1) 18x16 cm  2) 6x6 cm | NewTom 5G (Quantitative Radiology, Verona, Italy) | 110 kV  9.6 mA  FOV:  1) 18x16 cm  2) 6x6 cm  Voxel size: 0.3mm | None. | A) gutta-percha  B) metal post | None. | A1) 0.86  A2) 1,0  B1) 1,0  B2) 0.95 | A1) 1,0  A2) 1,0  B1) 0.89  B2) 1,0 | The specificity of CBCT decreased with the presence of a pin in the large-FOV group, but not in the small-FOV group. |
| Nascimento et al. 2014  Brazil | 40 teeth (molars) | UTM | Incomplete | According to the optimization filters:  A) original images  B) sharpen mild  C) sharpen super mild  D) s9  E) sharpen  F) sharpen 3x3  G) angio sharpen medium 5x5  H) angio sharpen high 5x5  I) shadow 3x3 | i-CAT (Imaging Sciences International, Hatfield, PA) | 120 kVp  8 mA  FOV: 8x8 cm  Voxel size: 0.2mm | Sharpen Mild, Sharpen Super Mild, S9, Sharpen, Sharpen 3x3, Angio Sharpen Medium 5x5, Angio Sharpen High 5x5, and Shadow 3x3; XoranCAT software | no filling | Dry human mandible | A) 0.568  B) 0.722  C) 0.683  D) 0.788  E) 0.763  F) 0.73  G) 0.696  H) 0.789  I) 0.659 | A) 0.583  B) 0.682  C) 0.692  D) 0.702  E) 0.738  F) 0.698  G) 0.765  H) 0.762  I) 0.667 | No statistical differences were observed in the diagnosis of VRF  when using filters. |
| Neves et al. 2014  Brazil | 30 single-rooted teeth | UTM | a) Complete  b) Incomplete | According to the root canal conditions:  A) no filling  B) gutta-percha  C) fiber post  D) metal post  According to the imaging mode:  1) high-fidelity  2) high-resolution  3) high-speed  4) standard  According to the type of fracture:  a) Complete  b) Incomplete | Accuitomo 3D (J. Morita, Kyoto, Japan) | 90 kV  5 mA  FOV: 4x4 cm  Voxel size: 0.08mm | None. | A) no filling  B) gutta-percha  C) fiber post  D) metal post | Dry human mandible | **  a)  A1) 0.92  A2) 0.92  A3) 0.86  A4) 0.84  B1) 0.74  B2) 0.82  B3) 0.74  B4) 0.70  C1) 0.88  C2) 0.82  C3) 0.86  C4) 0.86  D1) 0.50  D2) 0.50  D3) 0.54  D4) 0.50  b)  A1) 0.76  A2) 0.74  A3) 0.52  A4) 0.76  B1) 0.38  B2) 0.34  B3) 0.36  B4) 0.26  C1) 0.60  C2) 0.56  C3) 0.58  C4) 0.48  D1) 0.28  D2) 0.36  D3) 0.36  D4) 0.36 | **  a)  A1) 0.90  A2) 0.92  A3) 0.86  A4) 0.84  B1) 0.76  B2) 0.72  B3) 0.66  B4) 0.66  C1) 0.84  C2) 0.90  C3) 0.88  C4) 0.78  D1) 0.58  D2) 0.62  D3) 0.58  D4) 0.68  b)  A1) 0.90  A2) 0.92  A3) 0.86  A4) 0.84  B1) 0.76  B2) 0.72  B3) 0.66  B4) 0.66  C1) 0.84  C2) 0.90  C3) 0.87  C4) 0.78  D1) 0.58  D2) 0.62  D3) 0.58  D4) 0.68 | The CBCT imaging modes had little  influence in the diagnosis of complete and incomplete VRFs, whereas the presence of intracanal material had greater impact on the diagnostic ability. |
| Nikbin et al. 2018  Iran | 60 single-rooted teeth (premolars) | Pin and screwdriver | Incomplete | According to the root canal conditions (n=60):  A) gutta-percha  B) gutta-percha + metal post  According to the imaging mode, and specimen positioning (n=60):  1) original images, central position  2) original images, peripheral position  3) MAR algorithm, central position  4) MAR algorithm, peripheral position | Promax 3D (Planmeca, Helsinki, Finland) | **Kilovoltage and milliamperage determined automatically.  FOV: 5x8 cm  Voxel size: 0.16mm | Planmeca MAR algorithm | A) gutta-percha  B) gutta-percha + metal post | Bovine rib sockets | *  A1) 0.578  A2) 0.5773  A3) 0.7107  A4) 0.689  B1) 0.5777  B2) 0.3777  B3) 0.5333  B4) 0.333 | *  A1) 0.822  A2) 0.822  A3) 0.8443  A4) 0.8  B1) 0.622  B2) 0.6887  B3) 0.6443  B4) 0.7107 | Diagnostic accuracy was higher with central positioning than with peripheral positioning, irrespective of whether the MAR algorithm was applied. |
| Nikneshan et al. 2019  Iran | 62 teeth (premolars) | UTM | Incomplete | According to the imaging mode (n=62):  A) original images  B) MAR algorithm | Promax 3D (Planmeca, Helsinki, Finland) | 74 kVp  12 mA  FOV: 8x8 cm  Voxel size: 0.15mm | mild artifact reduction algorithm (Promax3d) | Root canal filling (gutta-percha and sealer) + metal post | Gypsum stone blocks | A) 0.742  B) 0.7206 | A) 0.8816  B) 0.8176 | The artifact reduction option creates no CBCT diagnostic difference in the presence of a post and does not provide better diagnosis of VRFs. |
| Oliveira et al. 2021  Brazil | 45 single-rooted teeth (premolars) | UTM | **Complete and incomplete (varied sample) | According to the root canal conditions:  A) no filling  B) gutta-percha  C) metal post  According to the imaging mode:  1) original images  2) MAR algorithm | OP300 Maxio (Instrumentarium Dental, Tuusula, Finland) | 90 kVp  10 mA  FOV: 6x4 cm  Voxel size: 0.085mm | OP300 MAR tool | A) no filling  B) gutta-percha  C) metal post | Dry human mandible | A1) 0.67  A2) 0.63  B1) 0.77  B2) 0.6  C1) 0.83  C2) 0.67 | A1) 0.87  A2) 0.8  B1) 0.67  B2) 0.77  C1) 0.53  C2) 0.23 | The OP 300 MAR tool negatively influenced the detection of VRFs in teeth with no root canal filling, gutta-percha, or metallic posts. |
| Özer, 2010  Turkey | 80 teeth (28 incisors, 28 premolars, 24 molars) | Hammer and chisel | Complete | According to the fracture thickness:  A) 0.2 mm  B) 0.4 mm  C) <0.2 mm | i-CAT (Imaging Sciences International, Hatfield, PA) | 120 kVp  3 mA  FOV: 4x4 cm  Voxel size: 0.125mm | None | No filling | Dry human mandible | NR | NR | CBCT scans are effective for detecting VRFs of different thicknesses. |
| Özer, 2011  Turkey | **60 single-rooted teeth (incisors, premolars and molars) | hammer and chisel | Complete | According to the voxel size:  A) 0.4 mm  B) 0.3 mm  C) 0.2 mm  D) 0.125 mm | i-CAT (Imaging Sciences International, Hatfield, PA) | 120 kVp  5 mA  FOV: 4x4 cm  Voxel size:  A) 0.4 mm  B) 0.3 mm  C) 0.2 mm  D) 0.125 mm | None. | No filling | Dry human mandible | A) 0.91  B) 0.93  C) 0.97  D) 0.98 | A) 0.93  B) 0.93  C) 0.96  D) 0.96 | CBCT scans were reliable  in detecting simulated VRF, and a 0.2-mm voxel was the  best protocol, considering the lower x-ray exposure and  good diagnostic performance. |
| Parrone et al. 2017  United States | 40 single-rooted teeth | Hammer and pin | Complete | According to the voxel size:  A) 0.075 mm  B) 0.1 mm  According to the imaging mode:  1) original images  2) optimization filter AINO | Promax 3D (Planmeca, Helsinki, Finland) | 90 kV  10 mA  FOV: 4x4 cm  Voxel sizes:  A) 0.075 mm  B) 0.1 mm | AINO (Adaptive Image Noise Optimiser) mode. | Root canal filling (gutta-percha) | Bovine rib sockets | A1) 0.97  A2) 0.96  B1) 1  B2) 0.95 | A1) 0.83  A2) 0.85  B1) 0.88  B2) 0.91 | The voxel size of 0.1 mm mode without filter is recommended for VRF detection in endodontically treated teeth. |
| Patel et al. 2013  United Kingdom | 28 teeth (14 premolars and 14 molars) | UTM | Complete and incomplete | According to the type of fracture:  A) Complete (n=40)  B) Incomplete (n=27)  C) overall (n=47) | Accuitomo 3D (J. Morita, Kyoto, Japan) | 90 kVp  3 mA  FOV: NR  Voxel size: 0.16mm | None. | Gutta-percha | Dry human mandible | A) 0.688  B) 0.533  C) 0.573 | A) 0.367  B) 0.367  C) 0.343 | The imaging artefacts caused by the gutta-percha root filling within the root canal most probably resulted in the overestimation of VRF with CBCT |
| Pinto et al. 2017  Brazil | 160 single-rooted teeth | Hammer and pin | **Complete and incomplete (mixed) | According to the root canal conditions (n=40):  A) no filling  B) gutta-percha  C) fiber post  D) metal post  According to the acquisition parameters (electric voltage and tube current) (n=40):  1) 74 kV/12 mA  2) 74 kV/10 mA  3) 74 kV/8 mA  4) 74 kV/6.3 mA  5) 70 kV/12 mA  6) 70 kV/10 mA  7) 70 kV/8 mA  8) 70 kV/6.3 mA | Kodak 9000 3D (Kodak Dental Systems, Carestream Health, Rochester, NY, EUA) | 1) 74 kV  12 mA  2) 74 kV  10 mA  3) 74 kV  8 mA  4) 74 kV  6.3 mA  5) 70 kV  12 mA  6) 70 kV  10 mA  7) 70 kV  8 mA  8) 70 kV  6.3 mA  FOV: 5x3.75 cm  Voxel size: 0.1mm | None. | According to the root canal conditions:  A) no filling  B) gutta-percha  C) fiber post  D) metal post | Dry human skull | A1) 73.55  A2) 76.50  A3) 76.50  A4) 76.50  A5) 76.50  A6) 76.50  A7) 79.45  A8) 76.50  B1) 70.60  B2) 70.60  B3) 61.75  B4) 64.70  B5) 70.60  B6) 61.75  B7) 55.85  B8) 58.80  C1) 76.50  C2) 79.45  C3) 79.45  C4) 76.50  C5) 79.45  C6) 76.50  C7) 79.45  C8) 73.55  D1) 58.80  D2) 58.80  D3) 58.80  D4) 52.90  D5) 61.75  D6) 61.75  D7) 58.80  D8) 58.80  Overall******:  A) 0.765  B) 0.6433  C) 0.7761  D) 0.5880 | A1) 87.00  A2) 87.00  A3) 89.15  A4) 91.35  A5) 89.15  A6) 89.15  A7) 89.15  A8) 89.15  B1) 91.30  B2) 84.80  B3) 87.00  B4) 84.80  B5) 87.00  B6) 89.15  B7) 91.30  B8) 89.15  C1) 87.00  C2) 89.15  C3) 86.95  C4) 89.15  C5) 89.15  C6) 89.15  C7) 84.80  C8) 87.00  D1) 82.65  D2) 87.00  D3) 84.80  D4) 84.80  D5) 87.00  D6) 87.00  D7) 87.00  D8) 78.30  Overall******:  A) 0.8889  B) 0.8806  C) 0.8779  D) 0.8482 | The variations in exposure parameters did not interfere with the diagnosis of VRF, independent of the root canal condition. Metallic posts influenced the diagnostic performance. |
| Pinto et al. 2021  Brazil | 40 single-rooted teeth | UTM | **Complete and incomplete (varied sample) | According to the acquisition parameters (mA, FOV):  A) 7 mA, 8x8 cm  B) 5 mA, 8x8 cm  C) 7 mA, 5x5.5 cm  D) 5 mA, 5x5.5 cm  According to the specimen position:  1) central  2) peripheral | Orthophos XG (Sirona, Bensheim, Germany) | 85 kVp  mA: NR  FOV: NR  Voxel size: 0.16mm | None. | root canal filling + metal post | Dry human skull. | *  A1) 0.55  A2) 0.4  B1) 0.575  B2) 0.425  C1) 0.725  C2) 0.625  D1) 0.75  D2) 0.65 | *  A1) 0.65  A2) 0.6  B1) 0.7  B2) 0.7  C1) 0.775  C2) 0.65  D1) 0.775  D2) 0.65 | Positioning the object in the center or closer to the anterior periphery of the FOV while using a small FOV improved the detection of VRF and decreased artifact  perception. |
| Queiroz et al. 2018  Brazil | 21 single-rooted teeth | UTM | Incomplete | According to the root canal condition (n=21):  A) no filling  B) gutta-percha  According to the acquisition parameters (FOV and voxel size)(n=21):  1) 4x4-cm FOV, 0.08-mm voxel  2) 10x10-cm FOV, 0.2-mm voxel  3) zoom reconstruction tool, 4x4-cm FOV, 0.08-mm voxel | Accuitomo 3D (J. Morita, Kyoto, Japan) | 80 kVp  6 mA  FOV: NR  Voxel size: NR | None. | A) no filling  B) gutta-percha | Dry human mandible | NR | NR | The Zoom Reconstruction  tool allows better accuracy for VRF detection in filled teeth, comparable to the high-resolution protocol. |
| Regan Anderson, 2017  United States | 60 single-rooted teeth (premolars) | Hammer and pin | Unclear | According to the root canal condition:  A) no filling  B) gutta-percha | CS 9000 3D (Carestream Dental, Rochester, NY) | 68 kVp  2 mA  FOV: NR  Voxel size: 0.076mm | None. | A) no filling  B) gutta-percha | Gypsum stone blocks | A) 0.947  B) 0.853 | A) 0.853  B) 0.773 | Limited-FOV CBCT is the most sensitive imaging modality for detection of VRFs among obturated and unobturated root samples. |
| Rosado et al 2024  Brazil | 22 mandibular premolars | UTM | Unclear | According to the FOV position:  a) anterior  b) posterior  According to the imaging mode:  A) standard  B) MAR algorithm  According to the metal post alloy:  1) Ag-Pd  2) Co-Cr  3) Ni-Cr | OP300 Maxio (Instrumentarium Dental, Tuusula, Finland) | 90 kVp  6.3 mA  FOV: 5x5 cm  Voxel size: 0.085mm | OP300 Maxio MAR algorithm | Metal posts:  1) Ag-Pd  2) Co-Cr  3) Ni-Cr | Dry human mandible | a)  A1) 0.44  A2) 0.44  A3) 0.48  B1) 0.62  B2) 0.60  B3) 0.54  b)  A1) 0.60  A2) 0.60  A3) 0.50  B1) 0.62  B2) 0.60  B3) 0.70 | a)  A1) 0.80  A2) 0.70  A3) 0.70  B1) 0.62  B2) 0.68  B3) 0.72  b)  A1) 0.72  A2) 0.63  A3) 0.65  B1) 0.55  B2) 0.53  B3) 0.57 | The MAR improved the sensitivity in VRF diagnosis. |
| Ruiz et al., 2024  Brazil | 20 single-rooted premolars | UTM | Unclear | According to the use of MAR algorithm:  A) Standard  B) MAR algorithm  According to the use of imaging filters:  1) no filter  2) Sharpen 1x  3) Sharpen 2x | OP300 Maxio (Instrumentarim Dental, Tuusula, Finland) | 90 kVp  8 mA  FOV: 5x5cm  Voxel size: 0.085mm | Algorith: OP300 Maxio metal artifact reduction algorithm (MAR)  Filters:  Sharpen 1x, Sharpen 2x, OnDemand 3D (Cybermed, Irvine, CA,  USA) | Metal post | Dry human mandible | A1) 0.53  A2) 0.56  A3) 0.51  B1) 0.58  B2) 0.76  B3) 0.69 | A1) 0.76  A2) 0.80  A3) 0.85  B1) 0.75  B2) 0.67  B3) 0.71 | MAR in conjunction with sharpening filters improved VRF detection. |
| Saati et al. 2019  Iran | 70 single-rooted teeth | Hammer and pin | Unclear | According to the CBCT device:  A) NewTom 3G  B) ProMax 3D  C) Cranex 3D  According to the imaging mode:  1) original images  2) MATLAB artifact removal software  3) MAR algorithm | A) NewTom 3G (Quantitative Radiology, Verona, Italy)  B) Promax 3D (Planmeca, Helsinki, Finland)  C) Cranex 3D (SoredexOy, Tuusula, Finland) | A) 90 kVp  10.65 mA  FOV: 15x15 cm  B) 84 kVp  14 mA  FOV: 8x8 cm  C) 110 kVp  4 mA  FOV: 6x8 cm  Voxel size: NR | 1) None  2) MATLAB artifact removal software  3) ProMax 3D and Cranex 3D MAR algorithm | Root canal filling (gutta-percha) | Bovine rib sockets | A1) 0.65  A2) 0.77  B1) 0.48  B2) 0.71  B3) 0.57  C1) 0.68  C2) 0.85  C3) 0.82 | A1) 0.65  A2) 0.71  B1) 0.51  B2) 0.62  B3) 0.60  C1) 0.68  C2) 0.80  C3) 0.70 | The MATLAB artifact removal software can enhance the detection of VRFs on CBCT scans to some extent. |
| Safi et al. 2015  Iran | 80 single-rooted teeth (premolars) | UTM | **Complete and incomplete (varied sample) | According to the CBCT device (n=80):  A) NewTom VGI  B) Scanora 3D | A) NewTom VG (Quantitative Radiology, Verona, Italy)  B) Scanora 3D (Soredex, Tuusula, Finland) | 90-110 kVp  12.5 mA  FOV:  A) 12x8 cm  B) 10x7.5 cm  Voxel size: 0.2mm | None. | Root canal filling (gutta-percha and sealer) + metal post (NiCr) | Acrylic blocks. | A) 0.638  B) 0.527 | A) 0.6706  B) 0.475 | The effect of metal artifacts on VRF detection was not significantly different between the two CBCT systems. |
| Safi et al. 2016  Iran | 80 single-rooted teeth (premolars) | UTM | Incomplete | According to the acquisition parameters (FOV and tube current)(n=80):  A) 10×7.5 mm FOV, 13 mA  B) 13×14.5 mm  FOV, 4 mA  C) 13×14.5 mm FOV, 13 mA | Scanora 3D (Soredex, Tuusula, Finland) | 90 kVp  A) FOV: 10×7.5 mm  13 mA  B) FOV: 13×14.5 mm  4 mA  C) FOV: 13×14.5 mm  13 mA  Voxel size: 0.25mm | None. | Root canal filling (gutta-percha and sealer) + metal post (NiCr) | Wax model | A) 0.7775  B) 0.6790  C) 0.7140 | A) 0.313  B) 0.667  C) 0.278 | A smaller FOV and lower mA should be preferably used for detection of VRFs in teeth with intracanal posts. |
| Salemi et al 2023  Iran | 20 single-rooted teeth | UTM | Unclear | According to the imaging mode:  A) standard  B) MAR algorithm  According to the distance to a dental implant:  1) 9mm  2) 20 mm | 9600 3D (Kodak Dental Systems, Carestream Health, Rochester, USA) | 60 kVp  8 mA  FOV: 8x5 cm  Voxel size: 0.075 | 9600 3D MAR algorithm | Root canal filling (gutta-percha + endodontic sealer) | Dry human mandible | *  A1) 0.575  A2) 0.875  B1) 0.525  B2) 0.725 | *  A1) 0.9  A2) 10  B1) 0.875  B2) 0.95 | Applying MAR algorithm had no positive effect on detection of VRFs on CBCT scans in both close and distant scenarios of implant position. |
| Shaker et al. 2019  Egypt | 120 single-rooted teeth | Rotation of a large post | Unclear | According to the application of MAR algorithm (n=120):  A) original images  B) SMAR mode (Scanora 3D) | Scanora 3D (Soredex, Tuusula, Finland) | 90 kVp  10 mA  FOV: 5x5 cm  Voxel size: 0.085mm | SMAR artifact algorithm, Scanora 3D | Root canal filling (gutta-percha + sealer) + metal post | None. | A) 0.65  B) 0.55 | A) 0.867  B) 1 | In the presence of metallic posts, CBCT with metal artifact reduction algorithm can improve detection of VRF. |
| Shokri et al 2022  Iran | 20 maxillary premolars | UTM | Unclear | According to the CBCT device:  a) CS 9300  b) Cranex3D  According to the milliamperage and voxel size:  a) CS 9300  A) 2 mA/0.09mm  B) 2 mA/0.2mm  C) 4 mA/0.09mm  D) 4 mA/0.2mm  b) Cranex3D  A) 4 mA/ 0.136mm  B) 4 mA/0.2mm  C) 10 mA/ 0.136mm  D) 10 mA/0.2mm  According to the intracanal posts:  1) brass  2) titanium  3) fiberglass  4) stainless steel  5) nickel-chromium | a) CS 9300 (Carestream Health, Rochester, USA)  b) Cranex3D (SoredexOy, Tuusula, Finland) | 90 kVp  FOV:  a) CS 9300  5x5 cm  b) Cranex3D  4x6 cm | None. | Metal posts:  1) brass  2) titanium  3) fiberglass  4) stainless steel  5) nickel-chromium | Dry human skull | NR | NR | Changes in amperage and voxel size within assessed values do not seem to influence the detection of VRF. Different intracanal post-materials have significant effect on VRF detection. |
| Taglihoo et al. 2018  Iran | 50 single-rooted teeth | Unclear | Unclear | According to the CBCT view:  A) axial  B) cross-sectional | NewTom 5G (Quantitative Radiology, Verona, Italy) | NR | None. | no filling | Gypsum stone blocks | A) 0.32  B) 0.2 | A) 1  B) 1 | The sensitivity, specificity, and accuracy of digital radiography and CBCT were not significantly different. |
| Takeshita et al. 2014  Brazil | 20 teeth | UTM | Unclear | According to the root canal conditions:  A) gutta-percha  B) gutta-percha + metal post | i-CAT (Imaging Sciences International, Hatfield, PA) | kVp: NR  mA: NR  FOV; 6X6 cm  Voxel size: 0.125mm | None. | A) gutta-percha  B) gutta-percha + metal post | Dry human mandible | NR | NR | CBCT was more accurate than conventional periapical radiography in detecting VRF. |
| Takeshita et al. 2015  Brazil | 20 teeth (premolars) | UTM | Unclear | None. | i-CAT (Imaging Sciences International, Hatfield, PA) | 120 kVp  mA: NR  FOV: 6x6 cm  Voxel size: 0.125mm | None. | No filling | Dry human mandible | NR | NR | CBCT showed the best results in the diagnosis of VRF. |
| Taramsari et al. 2013  Iran | 78 single-rooted teeth (premolars) | Hammer and pin | **Complete and incomplete (varied sample) | According to the root canal conditions:  A) gutta-percha  B) gutta-percha + fiber post  C) gutta-percha + metal post  According to the imaging mode:  1) high-resolution  2) standard | NewTom 5G (Quantitative Radiology, Verona, Italy) | A) 110 kVp  3.07 mA  FOV: 15x15 cm  Voxel size: 0.125-0.150 mm  B) 110 kVp  2.05 mA  FOV: 10x10 cm  Voxel size: 0.2-0.24 mm | None. | A) gutta-percha  B) gutta-percha + fiber post  C) gutta-percha + metal post | Macerated bone artificial sockets | A1) 0.8661  A2) 0.6923  B1) 0.6923  B2) 0.4615  C1) 0.6153  C2) 0.6153 | A1) 0.2307  A2) 0.3076  B1) 0.5384  B2) 0.3076  C1) 0.6153  C2) 0.50 | There were no significant differences between the diagnostic values of the two imaging modes used in the diagnosis of VRF or in the presence of root canal restorations. |
| Tofangchiha et al. 2017  Iran | 80 single-rooted teeth | UTM | Complete | According to the imaging mode:  A) original images  B) MAR mode | Promax 3D (Planmeca, Helsinki, Finland) | 66kVp  8 mA  FOV: 8x8 cm  Voxel size: NR | Promax 3D MAR mode | Metal post | Acrylic blocks | A) 0.54  B) 0.57 | A) 0.61  B) 0.69 | The sensitivity and specificity and accuracy of CBCT with  or without using the metal artifact reduction algorithms were similar. |
| Uysal et al. 2020  Turkey | 83 single-rooted teeth (premolars) | Hammer and pin | Complete | According to the voxel size:  A) 0.125 mm  B) 0.2 mm  C) 0.25 mm  D) 0.3 mm  E) 0.4 mm  According to the imaging mode:  1) original images  2) MAR | i-CAT (Imaging Sciences International, Hatfield, PA) | 120 kV  5 mA  FOV: 8x8 cm  Voxel sizes:  A) 0.125 mm  B) 0.2 mm  C) 0.25 mm  D) 0.3 mm  E) 0.4 mm | i-CAT AR mode | Root canal filling (gutta-percha + sealer) | Bovine rib sockets | *  A1) 0.9445  A2) 0.9445  B1) 0.917  B2) 0.917  C1) 0.917  C2) 0.917  D1) 0.722  D2) 0.693  E1) 0.583  E2) 0.583 | *  A1) 0.968  A2) 0.968  B1) 0.915  B2) 0.957  C1) 0.957  C2) 0.957  D1) 0.979  D2) 0.979  E1) 1  E2) 1 | High-resolution CBCT images resulted in an increase in sensitivity and specificity for detection of VRFs. The use of MAR did not further improve its diagnostic potential. |
| Uzun et al. 2015  Turkey | 74 single-rooted teeth (mandibular premolars) | Hammer and pin | Unclear | According to the CBCT device, and FOV:  A) 3D Accuitomo 170,4x4-cm FOV  B) 3D Accuitomo 170, 6x6-cm FOV  C) NewTom 3G, 15x15-cm FOV  D) NewTom 3G, 22x22-cm FOV | 1) Accuitomo 3D (J. Morita, Kyoto, Japan)  2) NewTom 3G (Quantitative Radiology, Verona, Italy) | 65 kVp  2 mA  FOV:  A)4X4cm  B)6x6cm  C)15x15cm  D)22x22cm  Voxel sizes:  A) 0.08mm  B) 0.125mm  C) 0.16mm  D) 0.25mm | None. | Root canal filling (gutta-percha + sealer) | Dry human mandible | NR | NR | No significant differences were found among observers or voxel sizes, with high Az results reported for all groups. |
| Valizadeh et al. 2011  Iran | 120 single-rooted teeth | UTM | Unclear | None. | New Tom 3G (Quantitative Radiology, Verona, Italy) | 110 kVp  1.9 mA  FOV: 23x23 cm  Voxel size: 0.3mm | None. | Metal post | Acrylic blocks. | *  0.883 | *  0.933 | CBCT provided favorable results regarding the diagnosis of VRF in teeth with metal posts. |
| Valizadeh et al. 2015  Iran | **60 single-rooted teeth (premolars) | UTM | **Complete and incomplete (varied sample) | According to the specimen position  A) central  B) 3 o’clock  C) 6 o’clock  D) 9 o’clock  E) 12 o’clock | New Tom VG (Quantitative Radiology, Verona, Italy) | 110 kVp  Automatically adjusted mA  FOV: 15x15 cm  Voxel size: 0.2mm | None. | Root canal filling (gutta-percha + sealer) + metal post | Acrylic blocks. | A) 0.334  B) 0.282  C) 0.375  D) 0.384  E) 0.367 | A) 0.651  B) 0.634  C) 0.547  D) 0.534  E) 0.5 | The central position is suitable for detection of VRF in teeth with intra-canal posts due to significantly higher sensitivity at this position |
| Van Acker et al., 2024  Belgium | 85 maxillary central incisors | UTM | Incomplete | According to the root canal conditions:  A) no filling  B) root canal filling with bioceramic cement (Biodentine)  According to the CBCT acquisition settings:  1) Child setting, high resolution  2) Adult setting, high resolution | Promax 3D (Planmeca, Helsinki, Finland) | 96 kVp  Voxel size: 0.1mm  1) 8 mA  FOV: 4.2x5cm  2) 10 mA  FOV: 5x5cm | None. | A) no filling  B) root canal filling with bioceramic cement (Biodentine) | Dry human skull | A1) 0.16  A2) 0.07  B1) 0.05  B2) 0.05 | A1) 0.82  A2) 0.90  B1) 0.91  B2) 0.98 | The presence of Biodentine did not affect the diagnostic performance of CBCT to detect VRFs. The different acquisition settings also did not impact the diagnosis of VRF. |
| Vanderburg, 2010  United States | 50 posterior teeth | Hammer and pin | Unclear | None. | Galileos Comfort (Sirona  Bensheim, Germany) | 85 kVp  42 mA  FOV: NR  Voxel size: 0.15mm | None. | no filling | Dry human skulls. | 0.6 | 0.49 | Periapical radiographs are more accurate than the Sirona Galileos Comfort CBCT scanner for the detection of vertical root fractures. |
| Varshosaz et al. 2010  Iran | 100 single-rooted teeth | UTM | Incomplete | None. | Promax 3D (Planmeca, Helsinki, Finland) | 70 kVp  6 mA  FOV: 8x8 cm  Voxel size: 0.16mm | None. | No filling | Dry human mandible | NR | NR | CBCT was shown to be significantly better than conventional periapical radiography for diagnosis of VRF. |
| Vieira et al. 2020  Brazil | 20 birooted teeth (maxillary first premolars) | Hammer and pin | **Complete and incomplete (varied sample) | According to the conditions (lingual + buccal root canals):  A) no filling  B) gutta-percha + gutta-percha  C) gutta-percha + fiber post  D) gutta-percha + metal core  E) fiber post + fiber post  F) metal core + metal core  G) metal post + metal post  According to the CBCT device:  1) CS9000 3D  2) OP300 | 1) CS 9000 3D (Carestream Dental, Rochester, NY)  2) OP300 Maxio (Instrumentarium Dental, Tuusula, Finland) | 1) 90 kVp  8 mA  FOV: 5x3.75 cm  Voxel size: 0.076mm  2) 90 kVp  8 mA  FOV: 5x5 cm  Voxel size: 0.085mm | None. | A) no filling  B) gutta-percha + gutta-percha  C) gutta-percha + fiber post  D) gutta-percha + metal core  E) fiber post + fiber post  F) metal core + metal core  G) metal post + metal post | Dry human skull | A1) 0.80  A2) 0.95  B1) 0.55  B2) 0.65  C1) 0.80  C2) 0.90  D1) 0.85  D2) 0.85  E1) 0.85  E2) 0.90  F1) 0.80  F2) 0.75  G1) 0.85  G2) 1 | A1) 0.95  A2) 1  B1) 0.90  B2) 0.80  C1) 0.95  C2) 0.85  D1) 0.95  D2) 0.60  E1) 0.90  E2) 0.95  F1) 0.95  F2) 0.90  G1) 0.70  G2) 0.30 | CS 9000 3D presented better performance than OP300 on VRF detection of endodontically treated teeth. |
| Wanderley et al. 2017  Brazil | 20 single-rooted teeth | UTM | Incomplete | According to the acquisition mode:  A) high resolution/standard (512 basis images)  B) high resolution/high density (1024 basis images) | Prexion 3D (Yoshida Dental, Tokyo. Japan) | 90 kVp  4 mA  FOV: 5x5 cm  Voxel size: 0.1mm | None. | Root canal filling (gutta-percha + sealer) | Dry human mandible | A) 0.97  B) 0.97 | A) 0.83  B) 0.90 | Both high-resolution imaging protocols presented high accuracy in the detection of incomplete VRFs of endodontically teeth. |
| Wanderley et al. 2018  Brazil | 30 single-rooted teeth | UTM | **Incomplete | According to the root canal conditions:  A) no filling  B) gutta-percha  C) metal post  According to the tooth orientation:  1) perpendicular to the horizontal plane  2) parallel to the horizontal plane | Picasso Trio 3D imaging system (Vatech, Hwaseong, Republic of Korea) | 85 kVp  5 mA  FOV: 5x5 cm  Voxel size: 0.2mm | None. | A) no filling  B) gutta-percha  C) metal post | Dry human mandible | A1) 0.76  A2) 0.787  B1) 0.56  B2) 0.554  C1) 0.5  C2) 0.554 | A1) 0.947  A2) 0.893  B1) 0.693  B2) 0.592  C1) 0.615  C2) 0.5 | The orientation of the tooth in relation to the projection plane of the x-rays does not influence the detection of VRF Irrespective of the intracanal material. |
| Wanderley et al., 2021  Brazil | 30 single-rooted teeth | UTM | Incomplete** | According to the root canal conditions:  A) no filling  B) gutta-percha  C) metal post  According to the scans assessment:  1) conventional (single scan, teeth positioned perpendicular to the x-rays)  2) combined (two scans: teeth positioned perpendicular and parallel to the x-rays) | Picasso Trio 3D imaging system (Vatech, Hwaseong, Republic of Korea) | 85 kVp  5 mA  FOV: 5x5 cm  Voxel size: 0.2mm | None. | A) no filling  B) gutta-percha  C) metal post | Dry human mandible | A1) 0.933  A2) 0.955  B1) 0.531  B2) 0.716  C1) 0.466  C2) 0.622 | A1) 0.911  A2) 0.909  B1) 0.805  B2) 0.805  C1) 0.604  C2) 0.843 | The diagnostic accuracy in teeth with intracanal material was improved when the assessment combines images obtained  at 2 orientations. |
| Wang et al 2023  Brazil | 40 single-rooted teeth | UTM | Incomplete | According to the imaging mode:  A) standard  B) MAR algorithm  According to the root canal conditions:  1) no filling  2) root canal filling | OP300 (Instrumentarium, Tuusula, Finland) | Kilovoltage: NR  Milliamperage: NR  FOV: 5x5 cm  Voxel size: 0.086mm | OP300 MAR algorithm | 1) no filling  2) root canal filling (gutta-percha + endodontic sealer) | Acrylic blocks | A1) 0.80  A2) 0.60  B1) 0.40  B2) 0.55 | A1) 0.65  A2) 0.70  B1) 0.90  B2) 0.65 | The use of the MAR algorithm increased the diagnostic accuracy in the detection of incomplete VRF on images of unfilled teeth. |
| Yamamoto-Silva et al. 2018  Brazil | 30 single-rooted teeth | UTM | *Complete and incomplete (varied sample) | According to the CBCT device, and voxel size:  A) Eagle 3D  1) 0.16 mm  2) 0.1 mm  B) i-CAT  1) 0.2 mm  2) 0.125 mm | A) Eagle 3D (Dabi Atlante, Brazil)  B) i-CAT (Imaging Sciences International, Hatfield, PA) | A) 85 kVp  5 mA  FOV: 5x5 cm  Voxel size:  1) 0.16 mm  2) 0.1 mm  B) 120 kVp  5 mA  FOV: 8x8 cm  Voxel size:  1) 0.2 mm  2) 0.125 mm | None. | Root canal filling and metal post (NiCr) | Dry human mandible | A1) 0.458  A2) 0.733  B1) 0.357  B2) 0.530 | A1) 0.571  A2) 0.700  B1) 0.633  B2) 0.800 | Protocols with a smaller voxel size and field of view seemed to favor the detection of VRF in teeth with intracanal metallic posts. |
| Yamashita et al., 2021  Brazil | 60 mandibular premolars | UTM | Unclear | According to the root canal conditions:  A) no filling  B) root canal filling +fiberglass post  C) root canal filling + metal post | i-CAT Next Generation (Imaging Sciences International, Hatfield, PA) | 120 kVp  37.07 mA  FOV: 8x8 cm  Voxel size: 0.125mm | None. | A) no filling  B) root canal filling +fiberglass post  C) root canal filling + metal post | Dry human mandible | A) 0.85**  B) 0.4**  C) 0.75** | A) 1.0**  B) 0.85**  C) 0.1.0** | The presence of metal posts did not influence accuracy; however, the presence of fiberglass post reduced the diagnostic capacity of CBCT. |

* Estimated by the reviewers of this systematic review
** Information obtained by email contact with the corresponding author
