# Supplementary material for: Exploring Diagnostic Reliability of CBCT for Vertical Root Fractures: A Systematic Review and Meta-Analytical Approach
Source: Int J Dent. 2025 Jul 21;2025:8824867. doi: 10.1155/ijod/8824867 (PMC12303641; doi:10.1155/ijod/8824867)
Supplement: Supporting Information 3 — QUADAS-2 questions adapted to this systematic review of in vitro studies. [file 8824867.f3.docx]

**SUPPLEMENTARY MATERIAL 3** QUADAS-2 questions adapted to this systematic review of *in vitro* studies.

| **Domain 1: Patient selection** | |
| --- | --- |
| 1. **Risk of bias** |  |
| **Describe methods of patient selection:** | |
| - **Was a consecutive or random sample of patients enrolled?** | Yes/No/Unclear |
| - **Was a case-control design avoided?** | Yes/No/Unclear |
| - **Did the study avoid inappropriate exclusions?** | Yes/No/Unclear |
| - **Was the sample size calculated?** | Yes/No/Unclear |
| - **Were the specimens visually analyzed to detect fractures/cracks before the experiments?** | Yes/No/Unclear |
| **Could the selection of patients have introduced bias?** | RISK: LOW/HIGH/UNCLEAR |
| 1. **Concerns regarding applicability** |  |
| **Describe included patients (prior testing, presentation, intended use of index test and setting):** | |
| **Is there concern that the included patients do not match the review question?** | CONCERN: LOW/HIGH/UNCLEAR |
| **Domain 2: Index test(s) *(if more than 1 index test was used, please complete for each test)*** | |
| 1. **Risk of bias** |  |
| **Describe the index test and how it was conducted and interpreted:** | |
| - **Were the index test results interpreted without knowledge of the results of the reference standard?** | Yes/No/Unclear |
| - **If a threshold was used, was it pre-specified?** | Yes/No/Unclear |
| - **Was used any simulation of the conditions *in vivo* (i.e., inserting the specimens in the alveolus of a dry human skull or mandible)** | Yes/No/Unclear |
| **Could the conduct or interpretation of the index test have introduced bias?** | RISK: LOW/HIGH/UNCLEAR |
| 1. **Concerns regarding applicability** |  |
| **Is there concern that the index test, its conduct, or interpretation differ from the review question?** | CONCERN: LOW/HIGH/UNCLEAR |
| **Domain 3: Reference standard** | |
| 1. **Risk of bias** |  |
| **Describe the reference standard and how it was conducted and interpreted:** | |
| - **Is the reference standard likely to correctly classify the target condition?** | Yes/No/Unclear |
| - **Were the reference standard results interpreted without knowledge of the results of the index test?** | Yes/No/Unclear |
| - **Was used any auxiliar method to visualize the fracture line (i.e., magnification, application of dyes, transillumination, etc.)** | Yes/No/Unclear |
| **Could the reference standard, its conduct, or its interpretation have introduced bias?** | RISK: LOW/HIGH/UNCLEAR |
| 1. **Concerns regarding applicability** |  |
| **Is there concern that the target condition as defined by the reference standard does not match the review question?** | CONCERN: LOW/HIGH/UNCLEAR |
| **Domain 4: Flow and timing** | |
| 1. **Risk of bias** |  |
| **Describe any patients who did not receive the index test(s) and/or reference standard or who were excluded from the 2x2 table (refer to flow diagram):**  **Describe the time interval and any interventions between index test(s) and reference standard:** | |
| - **Was there an appropriate interval between index test(s) and reference standard?** | Yes/No/Unclear |
| - **Did all patients receive a reference standard?** | Yes/No/Unclear |
| - **Did patients receive the same reference standard?** | Yes/No/Unclear |
| - **Were all patients included in the analysis?** | Yes/No/Unclear |
| - **Was the inter and intraexaminer agreement assessed?** | Yes/No/Unclear |
| **Could the patient flow have introduced bias?** | RISK: LOW/HIGH/UNCLEAR |
