# Supplementary material for: Exploring Diagnostic Reliability of CBCT for Vertical Root Fractures: A Systematic Review and Meta-Analytical Approach
Source: Int J Dent. 2025 Jul 21;2025:8824867. doi: 10.1155/ijod/8824867 (PMC12303641; doi:10.1155/ijod/8824867)
Supplement: Supporting Information 4 — Excluded articles and reasons for exclusion. [file 8824867.f4.docx]

**SUPPLEMENTARY MATERIAL 4** Excluded articles and reasons for exclusion.

|  | **Author, Year** | **Reason for exclusion** |
| --- | --- | --- |
| **01** | Aristizabal-Elejalde et al. 2020 | 6 |
| **02** | Ashmawy et al. 2018 | 5 |
| **03** | De Lima Rezende et al. 2016 | 1 |
| **04** | de Souza Coutinho-Filho et al. 2012 | 6 |
| **05** | Dutra et al. 2017 | 4 |
| **06** | Elsaltani et al. 2016 | 5 |
| **07** | Gaêta-Araujo et al. 2021 | 3 |
| **08** | Gulibire et al. 2020 | 6 |
| **09** | Guo et al. 2019a | 5 |
| **10** | Guo et al. 2019b | 5 |
| **11** | González et al. 2022 | 4 |
| **12** | Jakobson et al. 2014 | 5 |
| **13** | Johari et al. 2016 | 3 |
| **14** | Johari et al. 2017 | 3 |
| **15** | Karteva et al. 2016 | 4 |
| **16** | Kim et al. 2020 | 6 |
| **17** | Mansini et al. 2010 | 4 |
| **18** | Mizuhashi et al. 2020 | 6 |
| **19** | Mora et al. 2007a | 3 |
| **20** | Mora et al. 2007b | 3 |
| **21** | Quintero-Álvarez et al. 2021 | 6 |
| **22** | Shah et al. 2018 | 4 |
| **23** | Strobel et al. 2017 | 6 |
| **24** | Tangari-Meira et al. 2017 | 4 |
| **25** | Tiepo et al. 2017 | 4 |
| **26** | Wenzel et al. 2009 | 4 |
| **27** | Andraws Yalda et al. 2020 | 4 |
| **28** | Yuan et al. 2020 | 4 |

1. Studies with primary human teeth, or animal teeth (n=1); 2. Studies that included teeth with incomplete root formation (n=0); 3. Studies that did not evaluate CBCT as the index test (n= 5); 4. Studies that did not investigate the diagnostic accuracy of VRF (n= 10); 5. Studies with fracture simulation not consistent with the real aspect of VRF (n= 5); 6. In vivo studies (n= 7); 7. Reviews, letters, case reports and case series (n= 0).
